# Orai, RyR, and IP_3_R channels cooperatively regulate calcium signaling in brain mid-capillary pericytes

**DOI:** 10.1038/s42003-023-04858-3

**Published:** 2023-05-06

**Authors:** Braxton Phillips, Jenna Clark, Éric Martineau, Ravi L. Rungta

**Affiliations:** 1 Department of Neuroscience, Université de Montréal, Montréal, QC Canada; 2 Department of Stomatology, Faculty of Dental Medicine, Université de Montréal, Montréal, QC H3C3J7 Canada; 3 Centre interdisciplinaire de recherche sur le cerveau et l’apprentissage, Université de Montréal, Montréal, QC Canada

**Keywords:** Neuro-vascular interactions, Glial biology

## Abstract

Pericytes are multifunctional cells of the vasculature that are vital to brain homeostasis, yet many of their fundamental physiological properties, such as Ca^2+^ signaling pathways, remain unexplored. We performed pharmacological and ion substitution experiments to investigate the mechanisms underlying pericyte Ca^2+^ signaling in acute cortical brain slices of PDGFRβ-Cre::GCaMP6f mice. We report that mid-capillary pericyte Ca^2+^ signalling differs from ensheathing type pericytes in that it is largely independent of L- and T-type voltage-gated calcium channels. Instead, Ca^2+^ signals in mid-capillary pericytes were inhibited by multiple Orai channel blockers, which also inhibited Ca^2+^ entry triggered by endoplasmic reticulum (ER) store depletion. An investigation into store release pathways indicated that Ca^2+^ transients in mid-capillary pericytes occur through a combination of IP_3_R and RyR activation, and that Orai store-operated calcium entry (SOCE) is required to sustain and amplify intracellular Ca^2+^ increases evoked by the GqGPCR agonist endothelin-1. These results suggest that Ca^2+^ influx via Orai channels reciprocally regulates IP_3_R and RyR release pathways in the ER, which together generate spontaneous Ca^2+^ transients and amplify Gq-coupled Ca^2+^ elevations in mid-capillary pericytes. Thus, SOCE is a major regulator of pericyte Ca^2+^ and a target for manipulating their function in health and disease.

## Abstract (web summary)

Pharmacology and imaging of pericytes expressing GCaMP6f uncovers the basis of Ca2+ signaling in mid-capillary brain pericytes.

## Introduction

The mural cells of the brain are multifunctional cells organized in a continuum along the arterio-venous axis of the cerebral vasculature. This continuum is grouped into distinct cell types, which includes smooth muscle cells on penetrating arterioles (aSMCs), pre-capillary sphincters and ensheathing pericytes (also named terminal SMCs) of the arteriole-to-capillary transition zone, pericytes of the mid-capillary bed (of mesh and thin-strand morphology – here collectively referred to as mid-capillary pericytes), and venule smooth muscle cells^1,2^. Although they share a common origin^3^ and similar nomenclature, these cell types have numerous morphological^4,5^, transcriptomic^6,7^, and functional differences^8,9^. For example, aSMCs and ensheathing pericytes fully encircle and exhibit near complete coverage of the endothelial tube, highly express alpha smooth muscle actin (α-SMA), and undoubtedly regulate cerebral blood flow (CBF)^9,10^. Mid-capillary pericytes have thin or mesh-like processes that run longitudinal to the vessel, express little to no α-SMA, and their role in controlling CBF in physiological contexts remains poorly defined. Nevertheless, blood flow control aside, mid-capillary pericytes have numerous functions in health and disease, such as blood–brain-barrier regulation^11–13^ neuroimmune regulation^14^, angiogenesis^15^, glial scar formation^16^, and suggested stem cell-like properties^17^. Yet, despite these important functions, the basic physiological properties of brain mid-capillary pericytes, including the mechanisms that regulate their Ca^2+^ signaling, remain understudied.

All mural cells exhibit spontaneous fluctuations in intracellular Ca^2+^, which are termed Ca^2+^ transients^9,18,19^. Whereas the mechanisms underlying calcium signaling and contraction in aSMCs have been studied in detail (e.g. refs. ^20–26^.), investigations into brain pericyte Ca^2+^ signaling mechanism are only beginning to emerge. In ensheathing-type pericytes, Ca2+ transients can be caused by transmembrane influx through voltage-gated Ca2+ channels (VGCCs) and inositol 1,4,5-trisphosphate receptor (IP3R) signaling that recruits the Ca2+ required for α-SMA-mediated contraction^27,28^. Mid-capillary pericytes also exhibit Ca^2+^ transients with similar properties in vivo and in vitro, which are confined to spatial microdomains and modulated by neuronal activity^9,18,29^. Interestingly, a recent study reported that in resting conditions mid-capillary pericyte Ca^2+^ transients were only minimally sensitive to the potent L-type VGCC blocker nimodipine but were largely inhibited by the non-selective ion channel blocker SKF-96365^18^, consistent with a prior study reporting reduced functional expression of VGCCs on distal retinal capillaries^30^. In addition to non-selective cation TRPC channels, SKF-96365 blocks several other Ca^2+^ channels that are highly expressed in mid-capillary pericytes such as T-type VGCCs^31^, and the Orai family of store-operated Ca^2+^ channels^32^. Mid-capillary pericytes also express a plethora of Gq-coupled receptors^7,33^, which when activated would be expected to trigger Ca^2+^ release from internal stores via IP_3_R signaling. Therefore, a more detailed investigation is required to understand the cellular mechanisms underlying Ca^2+^ signaling in mid-capillary pericytes, their voltage dependence, and the interplay between Ca^2+^ release from internal stores and transmembrane influx.

Here, we utilized pharmacology experiments on acute cortical brain slices from transgenic mice expressing a fluorescent calcium indicator, GCaMP6f, in brain mural cells expressing PDGFRβ (PDGFRβ-Cre;GCaMP6f mice) to delineate the mechanisms underlying Ca^2+^ signaling in brain pericytes. Surprisingly, in contrast to ensheathing type pericytes which we show exhibit Ca^2+^ transients that are partially dependent on VGCCs, mid-capillary pericyte Ca^2+^ signals are largely unaffected by inhibition of VGCCs. We show that mid-capillary pericyte Ca^2+^ transients are, instead, primarily mediated by an interplay between store-operated Ca^2+^ entry (SOCE) channels and release of Ca^2+^ from internal stores by IP_3_Rs and ryanodine receptors (RyRs). Underscoring the potential importance of pericyte SOCE channels, we further demonstrate that they amplify Gq-coupled [Ca^2+^]_i_ elevations evoked by the potent vasoconstrictor endothelin-1 (ET-1).

## Results

**Fig. 1:**
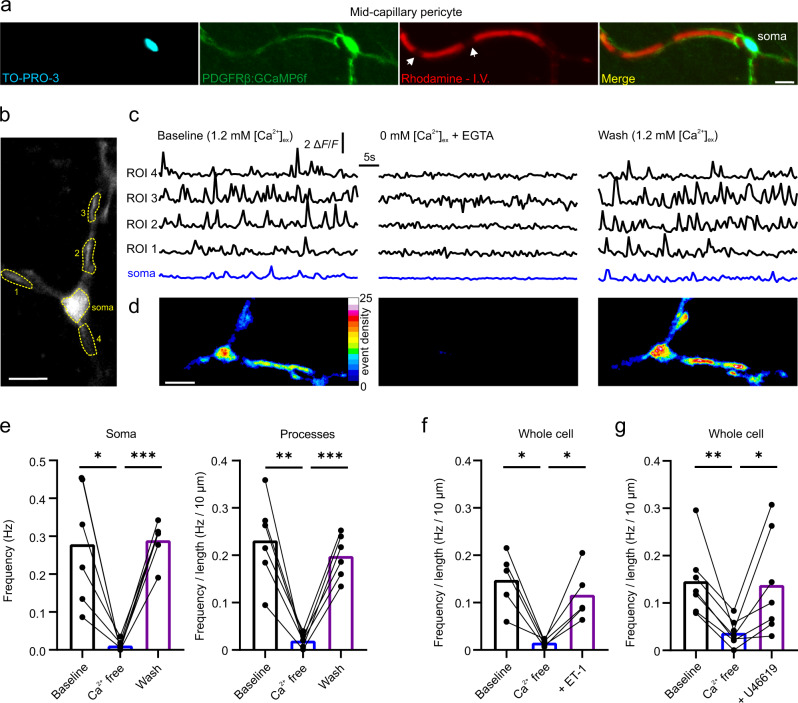
Imaging of mid-capillary pericyte Ca^2+^ transients and their dependence on extracellular Ca^2+^. **a** Confocal z-projection of a pericyte expressing GCaMP6f (green) in an acute brain slice loaded with TO-PRO-3 (cyan) and contacting a capillary whose lumen is labeled with RhodamineB-dextran (Red) – arrow heads indicate red blood cells. **b** Image of a GCaMP6f fluorescent pericyte and ROIs selected for **c**. **c** ROI based analysis of fluorescence changes in ROIs from (**b**) at baseline (left), after 11 min in Ca^2+^ free ACSF (middle), and 5 min following wash back of Ca^2+^ containing ACSF (right). **d** Heat maps of pericyte from (**b**, **c**) showing event density calculated from AQuA (percent time each pixel is active) in same conditions as above in **c**. **e** Summarized data showing effect of transiently removing and washing back [Ca^2+^]_ex_ on pericyte Ca^2+^ transient frequency in the soma and processes. Statistics were calculated by repeated measures one-way ANOVA followed by Tukey’s multiple comparisons test, baseline and wash were not significantly different. Analysis window of Ca^2+^ free (4–13 min), wash (4–13 min) following exposure to solution. *N* (mice) = 3; *n* (cells) = 6. **f**, **g** 10 nM ET-1 (**f**), or 100 nM U46619 (**g**) increases Ca^2+^ transient frequency after 10 min exposure to Ca^2+^ free ACSF. Statistics were calculated by repeated measures one-way ANOVA followed by Šídák’s multiple comparisons test. *N* = 2; *n* = 5 and *N* = 4; *n* = 7, respectively. For statistical comparisons *p < 0.05, **p < 0.01, ***p < 0.001. All image scale bars, 10 µm.

Pericyte Ca^2+^ signals were visualized with confocal imaging in acute cortical slices from PDGFRβ-Cre;GCaMP6f mice. The capillary lumen was labeled with an intravenous (I.V.) injection of RhodamineB-Dextran (70 kDa) and pericyte somas were labeled with the recently described pericyte specific dye TO-PRO-3^34^ (Fig. 1a). The spontaneous and spatially incoherent nature of mid-capillary pericyte Ca^2+^ transients led us to take advantage of the recently developed event-based analysis tool AQuA, which was developed precisely for unbiased analysis of such signals, but in astrocytes^35^. When applied to pericytes, AQuA was found suitable for extracting several properties of these pericyte Ca^2+^ transients, such as overall event frequency, amplitude, area, and duration (Fig. S1a–c; Supplementary Movie 1; and Tables S1 and S2). Indeed, Ca^2+^ transient frequency in mid-capillary pericyte somas as measured by AQuA was well-matched to measurements made with a region-of-interest based approach (Fig. 1b–d and S1d).

We first tested whether these Ca^2+^ transients depended on extracellular Ca^2+^ ([Ca^2+^]_ex_) by washing out [Ca^2+^]_ex_ from the perfused artificial cerebral spinal fluid (ACSF). Perfusion of 0 mM Ca^2+^, 2 mM EGTA solution reversibly depressed the frequency of Ca^2+^ transients in both the processes and soma of the pericyte (Fig. 1b–e and S1b–d). The inhibition of Ca^2+^ transients by Ca^2+^-free solution was rapid, reaching a maximal effect within 5–9 min (Fig. S1e). To test whether intracellular Ca^2+^ stores were completely depleted following 10 min in Ca^2+^-free ACSF, we applied Gq-coupled GPCR agonists for either the endothelin (ET)-A or thromboxane A2 receptor, ET-1 and U46619 respectively, which increase intracellular pericyte Ca^2+^ in an IP_3_-dependent manner. ET-1 and U46619 were still able to evoke Ca^2+^ transients in Ca^2+^-free solution (Fig. 1f, g), suggesting that Ca^2+^ stores were not fully depleted at this time point. However, after prolonged exposure to Ca^2+^ free solution (30 min) signals generated by ET-1 application were severely blunted when compared to those induced at 5 and 10 min following [Ca^2+^]_ex_ removal (Fig. S2), suggesting a gradual run down of store Ca^2+^ levels in the absence of transmembrane Ca^2+^ influx, and a dependence on [Ca^2+^]_ex_ to maintain ER store Ca^2+^. Intriguingly, in Ca^2+^ free solution the ET-1 and U46619 induced increases in Ca^2+^ transient frequency were temporary (ET-1, 248.2 ± 46.52 s; U46619, 268.6 ± 32.65 s) (Fig. S2a) in contrast to the sustained global elevation previously observed in normal [Ca^2+^]_ex_ solution^18^. Altogether, these results suggest mid-capillary pericyte Ca^2+^ transients require a plasmalemmal influx pathway, either to directly generate the transients or to sustain constitutive intracellular store filling and release.

**Fig. 2:**
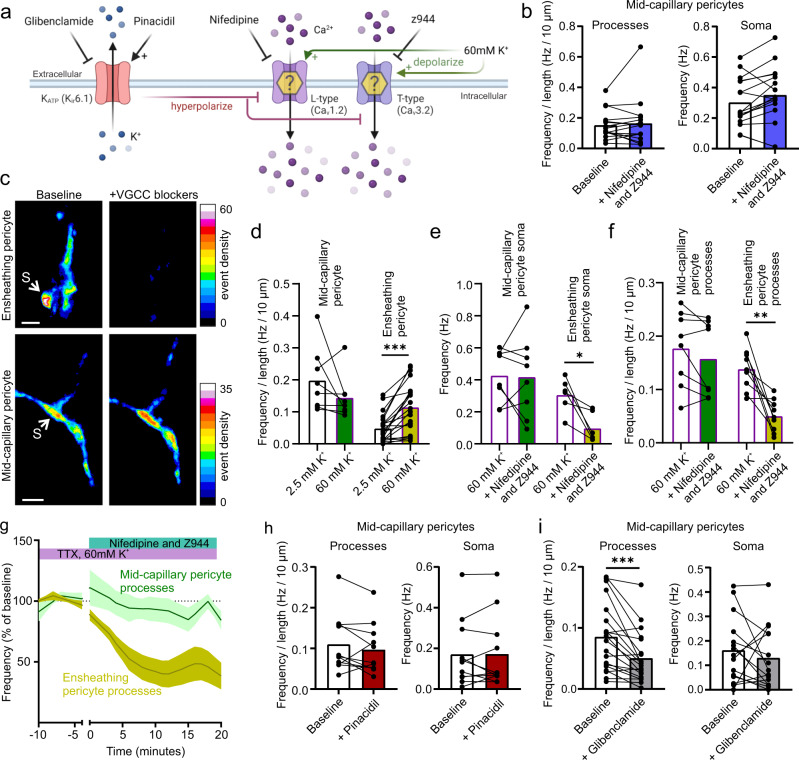
Mid-capillary pericyte Ca^2+^ transients are not potentiated by depolarization or mediated by VGCCs. **a** Schematic of experimental plan in Fig. 2. Created with biorender.com. **b** Blocking L-type and T-type VGCCs with 20 µM nifedipine and 2 µM Z944 does not reduce the frequency of Ca^2+^ transients in mid-capillary pericyte processes (*N* = 2, *n* = 16) or soma (*N* = 2, *n* = 16). Analysis was conducted 10–20 min after drugs reached the bath. **c** Example images showing event density (percentage of frames an event was detected within each pixel) after application of 20 µM nifedipine and 2 µM Z944 in 60 mM [K^+^]_ex_, for an ensheathing pericyte (3rd order, top), and a mid-capillary pericyte (bottom). S points to soma. **d** Summarized data showing that changing [K^+^]_ex_ from 2.5 to 60 mM does not increase the frequency of Ca^2+^ transients in mid-capillary pericytes (left, *N* = 2, n = 8), but does significantly increase Ca^2+^ transient frequency of ensheathing pericytes (1st–3rd order, *N* = 2, *n* = 21). Analysis done 9–18 min following 60 mM [K^+^]_ex_ exposure. **e** Summarized data, showing application of 20 µM nifedipine and 2 µM Z944 in 60 mM [K^+^]_ex_ reduces Ca^2+^ transient frequency in ensheathing (*N* = 5, *n* = 6) but not mid-capillary pericyte soma (*N* = 4, *n* = 8). Analysis was conducted 10–20 min after drugs reached the bath. **f** Summarized data, showing application of 20 µM nifedipine and 2 µM Z944 in 60 mM [K^+^]_ex_ reduces Ca^2+^ transient frequency in ensheathing (*N* = 5, *n* = 9) but not mid-capillary pericyte processes (*N* = 4, *n* = 8). Analysis was conducted 10–20 min after drugs reached the bath. **g** Time course of data shown in **f**. **h** Summarized data of K_ATP_ channel opener 10 µM pinacidil on mid-capillary pericyte Ca^2+^ transient frequency in processes (*N* = 2, *n* = 11), and soma (*N* = 2, *n* = 11). Analysis was conducted 10–20 min after drugs reached the bath. **i** Summarized data of K_ATP_ channel blocker 20 µM glibenclamide on mid-capillary pericyte Ca^2+^ transient frequency in processes (*N* = 3, *n* = 20), and soma (*N* = 3, *n* = 15). Analysis was conducted 10–20 min after drugs reached the bath. All experiments performed in 500 nM TTX. Shaded area (**g**) represents SEM. Time 0 represents the start of the first 50 s acquisition in the presence of the new bathing solution (when the drug(s) are estimated to have reached the slice chamber). For statistical comparisons *p < 0.05, **p < 0.01, ***p < 0.001. All image scale bars, 10 µm.

In ensheathing pericytes, depolarization triggers transmembrane influx of Ca2+ via VGCCs, thereby increasing Ca2+ transient frequency and [Ca^2+^]_i_^27,28^. Therefore, we tested the effect of blocking the predominantly expressed pericyte VGCC subtypes, Ca_V_1.2 (L-type) and Ca_V_3.2 (T-type), with nifedipine and Z944^36^, respectively (Fig. 2a). Application of nifedipine (20 µM) and Z944 (2 µM) had no effect on the frequency of Ca^2+^ transients, or baseline Ca^2+^ levels, in either the processes or soma (Fig. 2b, S4, and Tables S1 and S2). However, we could not exclude the possibility that VGCC transients would become more apparent if the membrane were depolarized, as previously reported for ensheathing type pericytes^27^. Consistent with this previous report, Ca^2+^ transient frequency of ensheathing pericytes was robustly elevated by increasing extracellular K^+^ concentration from 2.5 mM to 60 mM and was significantly reduced in the presence of VGCC blockers (Fig. 2c–g and Tables S1 and S2, 1st to 3rd branch order from penetrating arteriole). In contrast, depolarization with 60 mM [K^+^]_ex_ did not increase mid-capillary pericyte Ca^2+^ transient frequency (Fig. 2d) and VGCC blockers still had no effect on mid-capillary pericyte Ca^2+^ transient frequency when applied in 60 mM [K^+^]_ex_ (Fig. 2c–g and Tables S1 and S2). To further test the effect of membrane voltage on mid-capillary pericyte Ca^2+^, we pharmacologically altered K_ATP_ channel activity, whose modulation has been shown to have large effects on pericyte membrane potential^37–39^. Consistent with our above results showing that mid-capillary Ca^2+^ transients were independent of VGCCs, application of pinacidil (10 µM), a K_ATP_ channel opener which hyperpolarizes pericytes, had no effect on transient frequency in processes or the soma (Fig. 2h). Furthermore, glibenclamide (20 µM) a K_ATP_ channel blocker, decreased rather than increased transient frequency in mid-capillary pericyte processes (Fig. 2i), suggesting that mid-capillary K_ATP_ channels were open at rest, and that depolarization may actually decrease Ca^2+^ influx. As a note, all experiments were performed in tetrodotoxin (TTX, 500 nM) to block neuronal action potentials, which alone had no significant effect on the frequency of mid-capillary pericyte Ca^2+^ transients, consistent with a previous report^18^, although resting [Ca^2+^]_i_ was slightly increased in TTX (Figs. S3 and S4 and Tables S1 and S2). Taken together, these results indicate that in contrast to ensheathing pericytes which exhibit elevated Ca^2+^ transients upon depolarization via VGCCs, mid-capillary pericyte Ca^2+^ transients are largely independent of VGCCs.

**Fig. 3:**
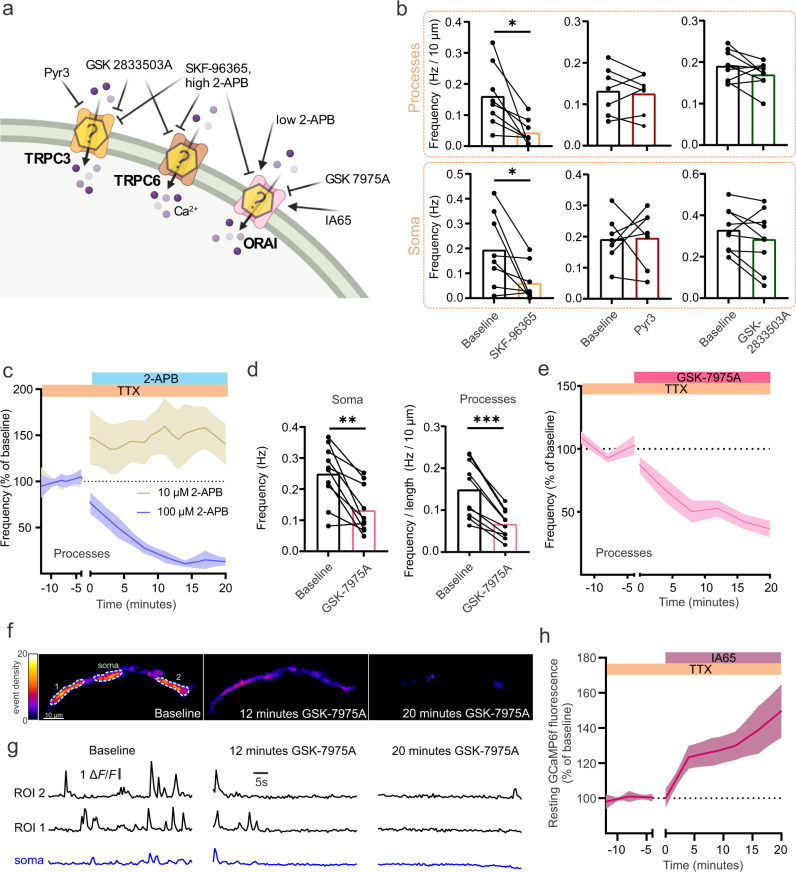
Mid-capillary pericyte Ca^2+^ transients signals are dependent on Orai Ca^2+^ channels. **a** Schematic of experimental plan and pharmacology. Created with biorender.com. **b** 100 µM SKF-96365 reduces the frequency of Ca^2+^ transients in mid-capillary pericyte processes (*N* = 3, *n* = 8) and soma (*N* = 3, *n* = 8). Neither of the TRPC3 or the TRPC3/6 antagonists, 20 µM Pyr3 or 10 µM GSK-2833503A, significantly reduced pericyte Ca^2+^ transient frequency in soma (*N* = 3, *n* = 7 and *N* = 2, *n* = 9, respectively) or processes (*N* = 3, *n* = 7 and *N* = 2, *n* = 9, respectively). Analysis was conducted 10–20 min after drugs reached the bath. **c** Low concentration (10 µM) 2-APB increases whereas high concentration (100 µM) 2-APB reduces Ca^2+^ transient frequency in mid-capillary pericyte processes (*N* = 3, *n* = 9 for both analyses). Analysis was conducted 10–20 min after drugs reached the bath. **d** The Orai channel inhibitor 40 µM GSK-7975A reduces Ca^2+^ transient frequency in both soma (left, *N* = 2, *n* = 10) and processes (right, *N* = 2, *n* = 10). Analysis was conducted 10–20 min after drugs reached the bath. **e** Time course of data in (**d** right). **f** Event density image of a pericyte showing a decrease in Ca^2+^ transients at different time points following application of GSK-7975A. **g** Example traces of GCaMP6f signal over time in ROIs from soma and 2 regions of a pericyte’s processes matching heat maps in **f**. **h** Perfusion of the Orai1/3 enhancer IA65 (10 µM) increases resting Ca^2+^ (GCaMP6f fluorescence measured in the soma, *N* = 2, *n* = 8). All experiments performed in 500 nM TTX. Shaded areas represent SEM. Time 0 represents the start of the first acquisition in the presence of the new bathing solution (when the drug(s) are estimated to have reached the slice chamber). For statistical comparisons *p < 0.05, **p < 0.01, ***p < 0.001.

Having established a marked difference in the mechanisms of Ca^2+^ signaling between pericyte subtypes, we next examined the unidentified calcium influx pathway(s) required for the generation of Ca^2+^ transients in mid-capillary pericytes (Fig. 3a). We first tested the non-specific ion channel blocker SKF-96365, which was previously reported to block mid-capillary pericyte Ca^2+^ transients^18^. Consistent with this previous report, Ca^2+^ transient frequency in the processes and somas of these pericytes was largely diminished in the presence of SKF-96365 (100 µM) (Fig. 3b and Tables S1 and S2). As SKF-96365 is a potent TRPC channel blocker, and TRPC3/6 are non-selective cation channels highly expressed in pericytes^7^, we further examined the sensitivity of mid-capillary pericyte Ca^2+^ transients to the TRPC3 blocker, Pyr3 and the potent TRPC3/6 blocker GSK-2833503A. Mid-capillary pericyte Ca^2+^ transients were unaffected by TRPC3 inhibition with Pyr3 (20 µM), and inhibition of TRPC3/6 with GSK-2833503A (10 µM) (Fig. 3b and Tables S1 and S2), suggesting influx via another SKF-96365 sensitive pathway. Interestingly, mid-capillary pericytes highly express Orai1 and Orai3 Ca^2+^ channels^7^, and these channels are also sensitive to SKF-96365^32^. We therefore tested the sensitivity of mid-capillary pericyte Ca^2+^ transients to the non-selective Orai channel blocker 2-APB. 2-APB is a potent Orai1 channel blocker at high concentration but potentiates Orai1 channels at low concentrations^40^. Consistent with the bidirectional concentration-dependent sensitivity of Orai1 channels to 2-APB, 10 µM 2-APB increased mid-capillary pericyte Ca^2+^ transient frequency, whereas 100 µM 2-APB nearly abolished all transients (Fig. 3c and Tables S1 and S2). Although the effects of 2-APB are supportive of Orai mediated Ca^2+^ entry, 2-APB has several off-target effects, such as inhibiting IP_3_Rs and some TRP channels. Therefore, we tested the sensitivity of mid-capillary pericyte Ca^2+^ transients to the Orai-specific blocker GSK-7975A^41^. Indeed, GSK-7975A (40 µM) robustly reduced the frequency of Ca^2+^ transients in mid-capillary pericytes in both the soma and processes (Fig. 3d–g and Tables S1 and S2). Additionally, we tested the effects of the recently developed compound IA65, which enhances Orai1/3 but inhibits Orai2^42,43^. Consistent with the high mRNA expression of Orai1 and Orai3 in mid-capillary pericytes, IA65 (10 µM) rapidly induced an elevation in resting Ca^2+^ levels (Fig. 3h and S4), although in contrast to 2-APB (10 µM), we did not observe an increase in Ca^2+^ transient frequency (Tables S1 and S2).

**Fig. 4:**
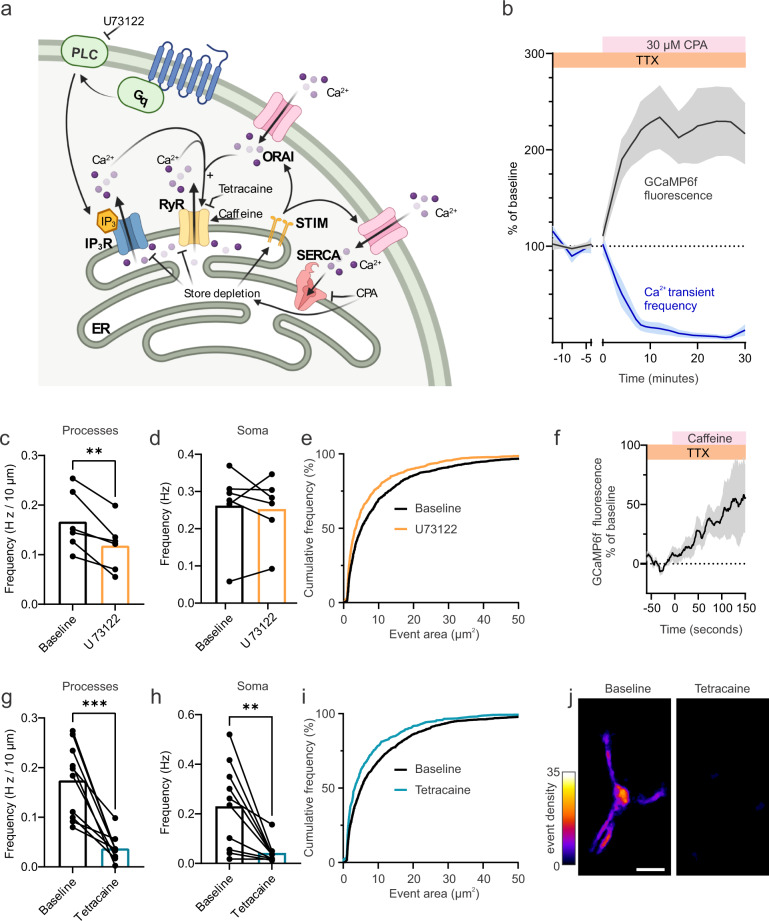
Mid-capillary pericyte Ca^2+^ transients are mediated by RyR and IP_3_R store release pathways. **a** Schematic of experimental plan and pharmacology. Created with biorender.com. **b** Perfusion of cyclopiazonic acid (CPA 30 µM) to block SERCA pump and deplete intracellular stores, reduces the frequency of mid-capillary pericyte Ca^2+^ transients (blue trace), while elevating cytosolic Ca^2+^ (black trace). *N* = 4, *n* = 9. **c**, **d** Effect of phospholipase C inhibition with U 73122 (25 µM) on mid-capillary pericyte Ca^2+^ transient frequency measured in the processes (**c**, *N* = 2, *n* = 6) and soma (**d**, *N* = 2, *n* = 6). Analysis was conducted 10–20 min after drugs reached the bath. **e** Cumulative frequency plot showing reduction in event area in the presence of U 73122. **f** Caffeine (10 mM) increases cytosolic Ca^2+^ in mid-capillary pericytes (GCaMP6f fluorescence measured in the soma). *N* = 4, *n* = 9. **g**, **h** Effect of RyR inhibition with tetracaine (200 µM) on mid-capillary pericyte Ca^2+^ transient frequency measured in the processes (**g**, *N* = 3, *n* = 10) and soma (**h**, *N* = 3, *n* = 10). Analysis was conducted 10–20 min after drugs reached the bath. **i** Cumulative frequency plot showing reduction in event area in the presence of tetracaine (200 µM). **j** Heat map of event density in a mid-capillary pericyte before and after application of tetracaine (200 µM). Scale bar, 10 µm. All experiments performed in 500 nM TTX. Shaded area (**b**, **f**) represents SEM. Time 0 represents the start of the first acquisition in the presence of the new bathing solution (when the solution is estimated to have reached the slice chamber). For statistical comparisons *p < 0.05, **p < 0.01, ***p < 0.001.

While our data suggests that Ca^2+^ influx through Orai SOCE channels is required for the generation of spontaneous Ca^2+^ transients in mid-capillary pericytes, store-operated entry and store-release mechanisms are inextricably linked. Therefore, we set out to test the dependence of mid-capillary pericyte Ca^2+^ transients on store Ca^2+^ filling and release pathways (Fig. 4a). If mid-capillary pericyte Ca^2+^ transients were dependent on store release, then blocking store filling would be expected to block the generation of the transients. Indeed, pharmacological inhibition of the sarco(endo)-plasmic reticulum calcium-ATPase (SERCA), with cyclopiazonic acid (CPA, 30 µM) abolished Ca^2+^ transients (Fig. 4b) suggesting their dependence on store release. In parallel to the drop in transient frequency, CPA induced a global and sustained resting Ca^2+^ elevation (Fig. 4b), consistent with the activation of Orai channels via ER Ca^2+^ depletion and STIM1/2 proteins. Given the dependence of the Ca^2+^ transients on store Ca^2+^, we proceeded to investigate their dependence on the ER store release channels, IP_3_Rs and RyRs. To test the relative contributions of these channels to mid-capillary pericyte Ca^2+^ transients, we either inhibited phospholipase C with U73122 (which prevents IP_3_ production) or RyRs with the reversible antagonist tetracaine. Blocking IP_3_ production with U73122 (25 µM) led a modest reduction in Ca^2+^ transient frequency in mid-capillary pericyte processes but not soma (Fig. 4c, d), and the spatial area of the remaining transients was also reduced (Fig. 4e). Strikingly, blocking RyRs with tetracaine (200 µM) robustly reduced the frequency, spatial area, and duration of Ca^2+^ transients in mid-capillary pericyte processes and soma (Fig. 4g–j and Tables S1 and S2), as well as resting Ca^2+^ levels (Fig. S4). We additionally confirmed the reversibility of the tetracaine effect on transient frequency in five cells (200–500 µM tetracaine = 30.33% ± 8.610%, washout = 108.2% ± 25.01% (% baseline frequency, mean ± SEM); *p* = 0.0305, two-tailed paired Students’ *t* test). Consistent with a functional role of RyRs in mid-capillary pericytes, application of caffeine (10 mM), a RyR potentiator, elevated mid-capillary pericyte Ca^2+^ (Fig. 4f). Altogether, these results point to a mechanism whereby constitutive Ca^2+^ entry through SOCE channels is required to maintain spontaneous Ca^2+^ release through RyRs and IP_3_Rs, which in turn lead to store depletion and activation of Orai channels.

**Fig. 5:**
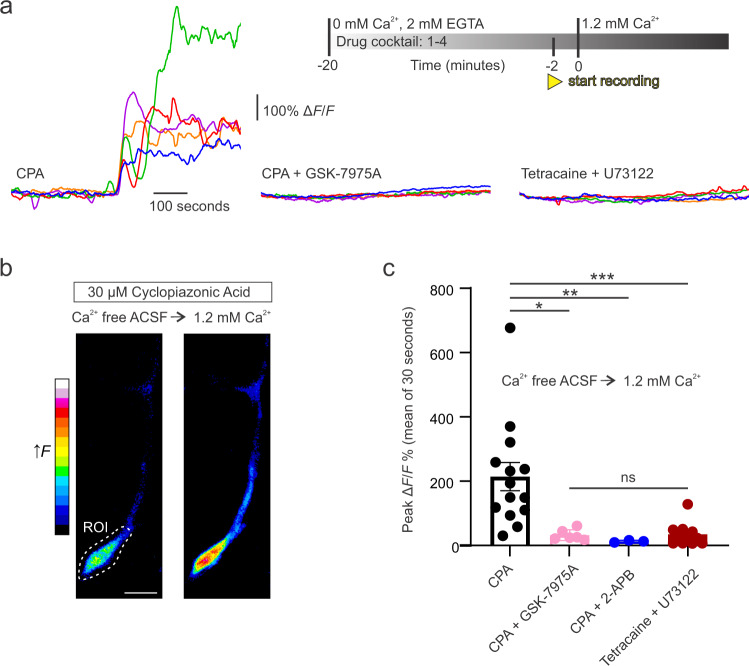
Mid-capillary pericytes express functional SOCE, that is blocked with Orai inhibitors. **a** Inset (top right), shows timing of experimental protocol. Five overlayed representative traces in different colors are shown in three different experimental conditions (left to right) from example cells in which 1.2 mM [Ca^2+^]_ex_ is washed back on the brain slice. Left, in 30 µM cyclopiazonic acid (CPA) to deplete ER stores and quantify the magnitude of Ca^2+^ entry via store-operated channels. Middle, same as left, but in the presence of the Orai inhibitor 40 µM GSK-7975A. Right, wash back of Ca^2+^ in condition where store depletion is prevented by blocking RyR and IP_3_R release pathways with tetracaine (200 µM) and U73122 (25 µM), respectively. **b** Example image showing increase in pericyte GCaMP6f fluorescence when extracellular ACSF is switched from Ca^2+^ free to 1.2 mM [Ca^2+^]_ex_ in conditions where endoplasmic reticulum stores are depleted with 30 µM CPA. An ROI around soma is used for quantification in **a** and **c**. Scale bar, 10 µM. **c** Summarized data showing effect of ACSF (*N* = 8, *n* = 14), 40 µM GSK-7975A (*N* = 2, *n* = 6), and 100 µM 2-APB (*N* = 1, *n* = 3) on SOCE, following transition from 0 mM to 1.2 mM Ca^2+^ ACSF in the presence of 30 µM CPA, and when store depletion is prevented by 200 µM tetracaine and 25 µM U 73122 (*N* = 2, *n* = 11). Statistics were calculated with a Kruskal-Wallis test followed by Dunn’s multiple comparisons test. Error bars represent SEM. For statistical comparisons *p < 0.05, **p < 0.01, ***p < 0.001.

To confirm that the Ca^2+^ influx through Orai channels was indeed store-operated, we tested whether Ca^2+^ entry to maximal store depletion was also sensitive to Orai channel antagonists, by adapting a protocol commonly used to maximally activate SOCE in expression systems and cell culture^44^. First, we applied CPA (30 µM) for 20 min in Ca^2+^-free ACSF to deplete ER stores and maximally activate “store-operated” channels. Then, we reintroduced 1.2 mM [Ca^2+^]_ex_ to the bathing solution while imaging a mid-capillary pericyte (Fig. 5a, b), triggering a large influx of Ca^2+^ through the open transmembrane channels. As expected, a large increase in mid-capillary pericyte Ca^2+^ was measured upon reintroduction of [Ca^2+^]_ex_, indicative of functional store-operated Ca^2+^ channels (Fig. 5a–c). As with the Ca^2+^ transients, this store depletion-induced Ca^2+^ influx was also sensitive to the Orai channel blockers, 2-APB and GSK-7975A, in mid-capillary pericytes (Fig. 5a, c). However, this experiment alone does not prove that store Ca^2+^ levels dictate the opening of the plasma membrane Orai channels, as the increase in cytoplasmic Ca^2+^ upon reintroduction of [Ca^2+^]_ex_ could simply be occurring through store independent channels that are constitutively open. Therefore, we performed an experiment in which the brain slice was exposed to Ca^2+^-free solution for the same duration as in the CPA experiments outlined above, but in the presence of tetracaine and U73122 to block store release via RyRs and IP3Rs. If plasma membrane Ca^2+^ influx is truly controlled by ER store Ca^2+^ levels in mid-capillary pericytes, then the increase in [Ca^2+^]_i_ upon reintroduction of [Ca^2+^]_ex_ should be smaller than when stores are depleted and SOCE is activated. Indeed, in slices perfused with 200 µM tetracaine and 25 µM U73122, re-introduction of [Ca^2+^]_ex_ resulted in a minute increase in Ca^2+^ entry that was not significantly different than when stores were depleted in the presence of Orai inhibitors (Fig. 5a, c). These results confirm that plasma membrane Ca^2+^ influx in mid-capillary pericytes is driven by ER store depletion.

**Fig. 6:**
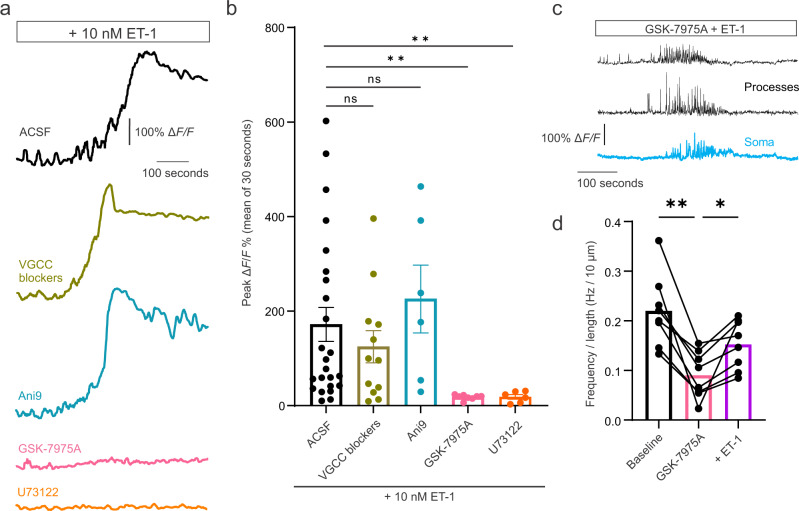
Ca^2+^ influx via Orai SOCE is required to sustain and amplify endothelin-1 induced [Ca^2+^]_i_ elevations. **a**, **b** Example traces (**a**) and summarized data (**b**) in different experimental conditions showing ET-1 evokes large amplitude and sustained Ca^2+^ elevations when applied alone (*N* = 10, *n* = 24), and which is not dependent on VGCC channels (blocked with 20 µM nifedipine + 2 µM Z944; *N* = 3, *n* = 12) or the Ca^2+^ activated Cl^-^ channel, TMEM16A (blocked with 2 µM Ani9; *N* = 3, *n* = 6). Blocking Orai Ca^2+^ channels with 40 µM GSK-7975A reduces the ET-1 evoked rise in intracellular Ca^2+^ (*N* = 3, *n* = 8). Blocking phospholipase C with 25 µM U73122 prevents ET-1 induced Ca^2+^ elevation (*N* = 2, *n* = 6), confirming it is dependent on GqGPCR-PLC pathway. Traces in **a** are temporally filtered with a 5 s running average. **c** Example trace showing that ET-1 induces a temporary increase in transient frequency when Orai channels are blocked with GSK-7975A (example is from the pericyte with the largest observed change in frequency). **d** Summarized data showing Ca^2+^ transient frequency increased (over a ~3 min window), when ET-1 is added in the presence of GSK-7975A. Statistics were calculated with a Kruskal–Wallis test followed by Dunn’s multiple comparisons test. All experiments performed in 500 nM TTX. Error bars represent SEM. For statistical comparisons *p < 0.05, **p < 0.01, ***p < 0.001.

The above evidence supporting SOCE channels in mid-capillary pericytes raised the intriguing possibility that Orai channels may be required to amplify and sustain GPCR mediated Ca^2+^ elevations, following robust release of Ca^2+^ from internal stores, as has been reported in other cell types (e.g. ref. ^45^). Therefore, we harnessed the potent vasoconstrictor ET-1, which has been previously shown to induce large elevations in mid-capillary pericyte Ca^2+^^18^. Indeed, in 1.2 mM [Ca^2+^]_ex_, ET-1 triggered a robust and sustained, but highly variable, Ca^2+^ elevation in mid-capillary pericytes (Fig. 6a, b). In contrast to the previously described role of the Ca^2+^ activated chloride channel, TMEM16A, and VGCCs, which are required to amplify ET-1 mediated Ca^2+^ elevations in ensheathing type pericytes (1st–3rd branch orders)^28^, in mid-capillary pericytes, blocking VGCCs (20 µM nifedipine and 2 µM Z944) or TMEM16A (2 µM Ani9) for 20 min had no significant effect on the magnitude of the ET-1 mediated Ca^2+^ elevation (Fig. 6a, b). However, when Orai channels were blocked with GSK-7975A for 20 min prior to ET-1 application, the magnitude of this elevation in [Ca^2+^]_i_ was strongly reduced (Fig. 6a, b), and as in Ca^2+^-free ACSF (Fig. S2), ET-1 evoked a transient increase in event frequency rather than a sustained elevation (Fig. 6c, d). Importantly, these Ca^2+^ elevations were prevented by U73122 (25 µM, 20 min), confirming that ET-1 induced signals are initiated by the Gq-GPCR-PLC pathway (Fig. 6a, b). These results suggest that Orai mediated SOCE amplifies Gq-GPCR mediated Ca^2+^ elevations and may contribute to sustained increases in mid-capillary pericyte Ca^2+^ following the release of vasoconstrictive agents.

## Discussion

Here we demonstrate that spontaneous Ca^2+^ transients in mid-capillary brain pericytes are mediated by an interplay between plasma membrane SOCE channels and ER store release through RyRs and IP_3_Rs. Our results are suggestive of bidirectional coupling, whereby Orai transmembrane Ca^2+^ influx is required to sustain spontaneous Ca^2+^ release through RyRs and IP_3_Rs, which in turn lead to store depletion and activation of Orai SOCE. These mechanisms are difficult to study in isolation: SOCE is dependent on store depletion, while store release is dependent on ER luminal Ca^2+^ load and intracellular Ca^2+^ levels, which are both heavily dictated by SOCE channels. Nevertheless, our pharmacological evidence, in conjunction with the short duration (~1–3 s) and spatially propagating nature of the observed Ca^2+^ transients, are consistent with a model in which spontaneous store release through RyRs and IP_3_Rs is maintained by constitutive SOCE^46,47^. Orai channels are ideally suited due to their high Ca^2+^ selectivity and tight coupling with luminal [Ca^2+^] in the ER via STIM proteins. Orai Ca^2+^ influx may additionally be critical for RyR activation by recruiting the intracellular Ca^2+^ required for calcium-induced-calcium-release (CICR) (e.g., ^48^), a mechanism which classically depends on L-type VGCCs in cardiac muscle^49^ and in smooth muscle^21^.

Our study adds to a growing body of evidence that the mural cells of the cerebral vasculature represent a continuum, with morphological, molecular, and functional heterogeneity along the arterio-venous axis. Our work is largely in agreement with a previous study which found that Ca^2+^ transients in mid-capillary pericyte processes were independent of L-type VGCCs at rest. However, in slight contrast to our results with 20 µM nifedipine, a minimal decrease in Ca^2+^ transient frequency within the soma was reported with 100 µM nimodipine^18^. Here we extend on these results and further show that these transients are not increased by depolarization and are likewise also independent of low-voltage-activated T-type calcium channels (Ca_V_3.2), which pericytes robustly express^7^. These findings contrast with our initial hypothesis that these transients would require VGCC activity, which was based on our previous finding that [Ca^2+^]_i_ and Ca^2+^ transient frequency decreases in mid-capillary olfactory bulb pericytes following local increases in neuronal activity, although on a slower time scale than upstream ensheathing pericytes and SMCs^9^. While we show that VGCCs are not required for mid-capillary pericyte Ca^2+^ transients, they highly express both L- and T-type VGCCs^7^ and it is possible that these channels become inactivated at ~−20 mV when exposed to 60 mM [K^+^]_ex_. Therefore, we cannot conclude that these channels are entirely functionally absent, but rather that Ca^2+^ signaling in mid-capillary pericytes is dominated by other mechanisms, which we report here. Intriguingly, a concurrent study has reported that distal retinal pericytes do show nifedipine-sensitive Ca^2+^ elevations in response to 60 mM [K^+^]_ex_^50^. However, in contrast to SMCs and ensheathing pericytes, L-type VGCCs were not required for the Ca^2+^ elevation in retinal mid-capillary pericytes following increased intraluminal pressure^50^, consistent with our results exhibiting that Ca^2+^ transients in different subtypes of pericytes exhibit a differential dependence on VGCCs.

Our results in ensheathing pericytes are largely consistent with recent reports that have uncovered prominent roles of depolarization, L-type VGCCs, and Gq-GPCR-IP_3_R signaling in mediating their Ca^2+^ transients and [Ca^2+^]_i_^27,28^. Elucidating the molecular and functional differences between pericyte subtypes, including in the Ca^2+^ signaling mechanisms described here, remains an important task. While SOCE is universal across cell types, it is more prominent in non-excitable cells, perhaps due, in part, to the inhibition of VGCCs by the essential SOCE proteins STIM1 and STIM2^51,52^, raising the possibility that elevated STIM-plasma membrane interactions could have inhibitory effects on mid-capillary pericyte VGCCs. Another interesting possibility is differential inhibition by endogenous polyamines, such as spermine, which has been previously suggested to underlie the decreased functional expression of VGCCs in distal capillary pericytes of the retina^30^. How these signaling differences translate into functional differences remains another key point of future study.

Although our data suggests Ca^2+^ influx in mid-capillary pericytes occurs primarily via non-voltage-gated channels, this does not exclude a role for pericyte hyperpolarization in functional hyperemia. Several reports on pericyte membrane potential have been made in various preparations from different regions of the CNS, reporting mean resting membrane potentials of between −35 mV and −50 mV^10,28,37–39,50^. In pressurized intact retina preparations, pericytes across the vascular arbor are found more depolarized than smooth muscle cells at low pressure (−43 mV vs. −64 mV)^50^, suggesting pericytes have lower K^+^ permeability at rest. Opening of K_ATP_ channels increases K^+^ permeability of mid-capillary pericytes, and therefore causes a robust hyperpolarization^37–39^, which can then be propagated to upstream mural and endothelial cells^37,39^. Retrograde hyperpolarization is a robust phenomenon in the microvasculature which rapidly closes VGCCs on upstream SMCs and ensheathing pericytes, thereby decreasing intracellular Ca^2+^ and dilating these contractile cells to increase local cerebral blood flow^9,27,53–55^. Interestingly, we found that blocking K_ATP_ channels with glibenclamide led to a decrease in the frequency of Ca^2+^ transients, consistent with a decreased driving force for Orai mediated Ca^2+^ influx. This result suggests that K_ATP_ channels may be open at rest in pericytes from our brain slices, potentially due to increased adenosine tone which can open K_ATP_ channels via A_2A_ receptors^37,38^. Although modest increases in [K^+^]_ex_ to 10 mM were previously suggested to decrease Ca^2+^ transient frequency via K_ATP_ dependent hyperpolarization^18^, unlike K_ir_2.x channels, K_ATP_ channels are not known to be activated by [K^+^]_ex_. If K_ATP_ channels were indeed open at rest, as appears to be the case in our study, even modest increases in K^+^ would depolarize pericytes via these open K^+^ channels. Therefore, an alternative interpretation of these results could be that blocking K_ATP_ channels in their experiments blocked K^+^ induced depolarization rather than hyperpolarization, and thereby reduced driving force for Ca^2+^ entry via non-voltage-gated channels, consistent with our findings. Recordings of mid-capillary pericyte membrane potential changes in response to modest changes in K^+^ concentration are therefore needed to properly interpret these differences.

During neurovascular coupling, ensheathing pericytes of the capillary-arteriole transition zone (which robustly express α-SMA) dilate rapidly, whereas the pericytes of the mid-capillary bed (low or negative for α-SMA) increase their diameter more slowly^9,56^. Whereas ensheathing type pericytes dilate actively and independently from the arteriole^10^, it remains to be determined whether the mid-capillary diameter increase is a purely passive process or has an active component mediated by mid-capillary pericytes. Interestingly, under pathological conditions such as Alzheimer’s disease and stroke, even mid-capillary pericytes are found to be constricted^28,57^, and prolonged optogenetic activation of ChR2 on cortical mid-capillary pericytes has been shown to locally constrict the capillaries that they contact on a slow timescale^8,58^. Likewise, increases in intraluminal pressure lead to mid-capillary pericyte depolarization in the retina, and are followed by a delayed constriction^50^. Although these studies suggest that mid-capillary pericyte membrane depolarization is correlated with increased rigidity and tone of the capillary bed, a causal relationship is lacking. Given that our data suggests that mid-capillary pericyte Ca^2+^ influx is dominated by voltage-independent channels, depolarization would be expected to reduce this Ca^2+^ influx and subsequent store filling, due to a decrease in driving force, as in other non-excitable cells. This is consistent with a recent report showing that hyperpolarization of mid-capillary pericytes by application of pinacidil increased pressure-induced Ca^2+^ influx^50^.

Finally, we show here that SOCE is required to amplify the mid-capillary pericyte Ca^2+^ elevation mediated by the vasoconstrictor ET-1. The magnitude of the ET-1-induced Ca^2+^ responses when applied in normal ACSF were highly variable, which could reflect heterogeneous expression of the endothelin-A receptor and/or SOCE proteins in mid-capillary pericytes. ET-1 is a central molecule to pericyte pathology, which is released in ischemia^59^, and which is elevated downstream of Amyloid-β oligomers to constrict pericytes in Alzheimer’s disease^57^. In ensheathing pericytes (1–3rd branch order), the ET-1 evoked [Ca^2+^]_i_ increase was also recently shown to require an amplification step, but via Ca^2+^ activated Cl^-^ channel mediated depolarization and L-type VGCC activation^28^. Our results indicate a similar amplification process in mid-capillary pericytes, but with a clear molecular divergence in the mechanisms mediating ET-1 evoked Ca^2+^ influx. As Ca^2+^ is a ubiquitous signal transduction molecule throughout biology, mediating an array of cellular functions, including gene transcription and contraction, SOCE channels may therefore play an important role in numerous pericyte functions and contribute to their dysfunction in disease.

### Study limitations

Genetic tools will ultimately be required to uncover the precise molecular identities underlying mid-capillary pericyte Ca^2+^ signaling. Our pharmacological modulation of SOCE with non-selective (SKF-96365 and 2-APB) and relatively selective (GSK-7975A and IA65) compounds is consistent with the highly Ca^2+^ selective family of Orai channels (namely, Orai1 and Orai3) as the molecular identities underlying pericyte SOCE. However, TRPC channels have been shown to interact with and contribute to SOCE in certain conditions^60,61^, although this concept is heavily debated^62^. While our results show that TRPC3/6 are not major contributors, we cannot completely rule out participation of the TRPC1/4/5 subfamily to pericyte SOCE.

The molecular identities of the IP_3_R and RyR channels in mid-capillary pericytes will similarly require genetic tools to elucidate. While the IP_3_Rs are broadly dispersed throughout cell types, the distribution of RyR isoforms is more segregated, with RyR2 constituting the dominant variant in smooth muscle cells^63^, making it the most likely variant to be functional in pericytes. Interestingly, RyRs have been classified as functionally absent in most^64^ (but not all^65,66^) pericytes of different tissues, including ensheathing type pericytes in the retinal CNS vasculature^27^. It remains to be determined whether the functional expression of RyRs in cortical CNS mid-capillary pericytes represents a difference across brain regions and/or between pericyte subtypes.

Our study was conducted solely in the brain slice preparation, which is a highly useful tool for probing cellular signaling mechanisms, as pharmacological compounds can be delivered at a controlled concentration and cellular architecture is kept largely intact. However, there are important limitations when compared to in vivo settings: there is no blood pressure and myogenic tone, arterioles and capillaries are collapsed, oxygen concentration (95%) is supraphysiological, and the slice surface has undergone mechanical damage. Translation of this work to in vivo experimentation is an important future direction.

## Methods

### Ethics statement and animals

All procedures conformed to the guidelines of the Canadian Council on Animal Care and were approved by the “Comité de déontologie sur l’expérimentation animale” (CDEA) of the Université de Montréal (QC, Canada). PDGFRβ-Cre mice^67^ were crossed with Ai95(RCL-GCaMP6f)-D reporter mice (Jackson Laboratory) to obtain PDGFRβ-Cre; GCaMP6f-floxed double transgenic mice. Male and female mice aged P28-P163 were used in experiments.

### Acute brain slice preparation

Prior to slicing, mice were put into deep anesthesia with isoflurane and 50 µL of Rhodamine B isothiocyanate-Dextran (70 kDa, 2.5% wt:vol, Sigma-Aldrich) was injected retro-orbitally to label the vessel lumen. Mice were euthanized by decapitation. Following extraction from the skull, brains were placed into ice-cold NMDG-based slicing solution containing (in mM): 120 *N*-Methyl-d-glucamine, 3 KCl, 25 NaHCO_3_, 7 MgCl_2_-6H_2_O, 1 NaH_2_PO_4_-H_2_O, 20 Glucose, 2.4 Na-pyruvate, 1.3 Na-ascorbate, 1 CaCl_2_-H_2_O. Then, 300 µm thick coronal cortical slices were cut with a Leica VT 1200S Vibratome. Slices were transferred to a custom chamber with artificial cerebral spinal fluid (ACSF) containing (in mM): 126 NaCl, 2.5 KCl, 26 NaHCO_3_, 1.5 MgCl_2_-6H_2_O, 1.3 NaH_2_PO_4_-H_2_O, 10 Glucose, and 1.2 CaCl_2_ at 36 °C for 10 min. Slices were then recovered in the chamber at room temperature until use. The fluorescent dye TO-PRO-3 has previously been shown to robustly label mid-capillary pericytes in fixed tissue^34^, and we have adapted this for imaging in acutely prepared live brain slices. Prior to use, slices were incubated in 1 µM TO-PRO-3 diluted in ACSF for 20 min at room temperature to label and identify the pericyte soma. All solutions used were continuously gassed with 95% O_2_ and 5% CO_2_.

### Pharmacology and ion substitution

All salts were obtained from Sigma-Aldrich. Please refer to Table S3 for identifiers, and vehicles of reagents used. Time 0 represents the start of the first acquisition when the new solution is estimated to have reached the bath (estimated based on the flow rate). For Ca^2+^-free ACSF, CaCl_2_ was omitted from the ACSF and 2 mM EGTA was added. For 60 mM K^+^ ACSF, equimolar NaCl was replaced with KCl. Slices were perfused with TTX for 10–20 min prior to imaging. For 60 mM K^+^ experiments, slices were perfused with 60 mM K^+^ for 10-20 min prior to application of VGCC blockers.

### Confocal imaging

Imaging was performed with a Zeiss LSM 510 laser scanning confocal microscope with a 40X water immersion objective lens (0.8NA). Pericytes in cortical brain slices were located by TO-PRO-3 and GCaMP6f co-localization and association with a Rhodamine B labeled vessel. GCaMP6f was excited with a 488 nm LED laser and was detected after passing through a 505–530 nm bandpass filter. TO-PRO-3 and Rhodamine B were excited with a 633 nm and 543 nm HeNe laser respectively, which were generally turned off during Ca^2+^ imaging. Ensheathing pericytes in a subset of explicitly labeled experiments were identified based on GCaMP fluorescence almost fully enwrapping the vessel and were definitively confirmed to be within 3 branch orders of the penetrating arteriole. Mid-capillary pericytes were selected for all other experiments on the basis of: (1) Morphology: thin-stand or mesh morphology in which processes clearly did not fully enwrap the vessel (notably different than those found on first 3 branches), (2) being positive for TO-PRO-3 labeling, and 3) the lack of an arteriole detected within 4 branches of the capillary using the Rhodamine B signal. In several cases for mid-capillary pericytes they could not be traced back 4 branches and an arteriole was not visible in the field of view, in these cases only criteria 1 and 2 were used. To measure calcium transient frequency over time, 50 s image acquisitions were made every 3–4 min in frame scanning mode at a 2 Hz sample rate. To record SOCE experiments and [Ca^2+^]_i_ rises evoked by agonists, a single acquisition was made in frame scanning mode at a 2 Hz sample rate. Slices were perfused with ACSF at 2 mL/min and were kept at 34 ± 2 °C during experiments.

### Data collection and analysis

Between frame XY-plane translational movement and within frame distortion were removed using a custom non-rigid alignment MATLAB algorithm, based on the *imregdemons* (AccumulatedFieldSmoothing = 2.5; PyramidLevels = 4; 32, 16, 8, and 4 iterations respectively for each pyramid level) and *imwarp* MATLAB functions (https://scanbox.org/2016/06/30/non-rigid-image-alignment-in-twenty-lines-of-matlab/). Hand-drawn regions of interest (ROI) separated pericyte somas from processes. The Astrocyte Quantification and Analysis (AQuA) MATLAB tool^35^ was used for unbiased event-based Ca^2+^ transient analysis. A Gaussian filter was applied to the images (*σ* = 2), minimum event size was set to (pixels): 5/pixel size (µm), and detected events occurring in the same frame separated by (in pixels) 1/pixel size (µm) were merged. Event threshold parameters were set to reliably detect the majority of pericyte Ca^2+^ signaling events with minimal detection of false events outside the cell boundaries. Identical analysis parameters were used for all time series of a given cell and experiment. In experiments in which movies were collected at differing timepoints and average data was shown, a linear interpolation between data points was performed. Ca^2+^ transients following agonist application (ET1 or U46619) in Ca^2+^ free solution (Fig. 1f, g) or GSK-7975A (Fig. 6c, d) were only momentarily increased after agonist application before stores became depleted and were therefore analyzed over a brief period during which the agonists visibly evoked an increase in transient signals: ET-1 in Ca^2+^-free (65.2 ± 16.31 s); U46619 (78.86 ± 12.53 s); ET-1 in GSK-7975A (149.1 ± 7.841 s). To compare ET-1 responses at 5, 10, and 30 min in Ca^2+^-free solution (Fig. S2), Ca^2+^ transient frequency following ET-1 application was analyzed over a 50 s timeframe from when the agonist first evoked signals. To examine changes in ensheathing pericyte Ca^2+^ transient frequency following application of 60 mM [K^+^]_ex_, an arteriole was located and single 50 s acquisitions of pericytes 1–3 branch orders downstream of the arteriole were taken before and 9–18 min after 60 mM [K^+^]_ex_ application. Ca^2+^ transient data during all other treatments in Figs. 2–4 was measured from 10–20 min after drug application unless otherwise stated. Transient frequency was normalized to 10 µm of pericyte processes (referred to as ‘length’). To measure SOCE and [Ca^2+^]_i_ rises evoked by ET-1 in Figs. 5–6, background fluorescence was subtracted, a hand-drawn ROI was placed around the soma, and Δ*F*/*F* was averaged 30 s around the peak value recorded. Event density heat maps in figures represent the fraction of frames in which an active event was present in each pixel. To measure resting Ca^2+^ levels (Fig. S4 and Table S1 and S2), a hand-drawn ROI was placed around the soma and an average fluorescence value was calculated from pixels in which AQuA did not detect an event for a given frame. Only cells in which the soma was in focus were included for this analysis. Five cells were excluded due to improper alignment of translational movement between recordings, and six cells were excluded as outliers following identification with the ROUT test (*Q* = 10%).

### Statistics

For all paired data with two groups a two-tailed paired Students’ *t* test was conducted. For paired data with three groups, a one-way repeated measures ANOVA was conducted, which if significant, was followed by Tukey’s or Šidák’s post-hoc test (see Figure Legends). For all other statistical comparisons normality was first assessed with a Shapiro–Wilk normality test. If this test failed, statistics were calculated by a Kruskal–Wallis non-parametric test, followed by Dunn’s multiple comparisons test.

### Software availability

Software used in the study is open source.

### Reporting summary

Further information on research design is available in the Nature Portfolio Reporting Summary linked to this article.

## Supplementary information

Supplementary Information

Description of Additional Supplementary Data

Supplementary Data 1

Supplementary Movie 1

Reporting Summary

## Data Availability

Source data is available with the manuscript as Supplementary Data 1.
