# Supplementary material for: Orai, RyR, and IP3R channels cooperatively regulate calcium signaling in brain mid-capillary pericytes
Source: Commun Biol. 2023 May 6;6:493. doi: 10.1038/s42003-023-04858-3 (PMC10164186; doi:10.1038/s42003-023-04858-3)
Supplement: Supplementary file 1 — Supplementary Information [file 42003_2023_4858_MOESM1_ESM.pdf]

Supplementary Information (Phillips et al., 2023)

Contents:

<u>Supplementary Figures 1-4</u>

Figure S1: Related to Fig. 1, Imaging of mid-capillary pericyte microdomain Ca^{2+} signals and their dependence on extracellular Ca^{2+}.

Figure S2: Related to Fig. 1, Effect of ET-1 at different time points following extracellular Ca^{2+} removal.

Figure S3: Related to Fig. 2-4, Analysis of Ca^{2+} transient frequency in response to DMSO and 500 nM TTX.

Figure S4: Related to Fig. 2-4, Effects of pharmacological compounds on resting Ca^{2+}.

<u>Supplementary Table 1-3</u>

Table S1: Summarized data and statistics for pharmacological treatments on pericyte processes and effect of 60 mM K^{+}.

Table S2: Summarized data and statistics for all pharmacological treatments on pericyte soma and resting Ca^{2+}.

Table S3: List of pharmacological compounds, intended targets, vehicles, and associated references.

<u>Supplementary Movie 1:</u>

Movie S1: Related to Fig. 1, the movie shows GCaMP6f fluorescence over a 50 second recording of a mid-capillary pericyte (left), and colour coded identification of events detected by AQuA (right).

Supplementary Figure 1

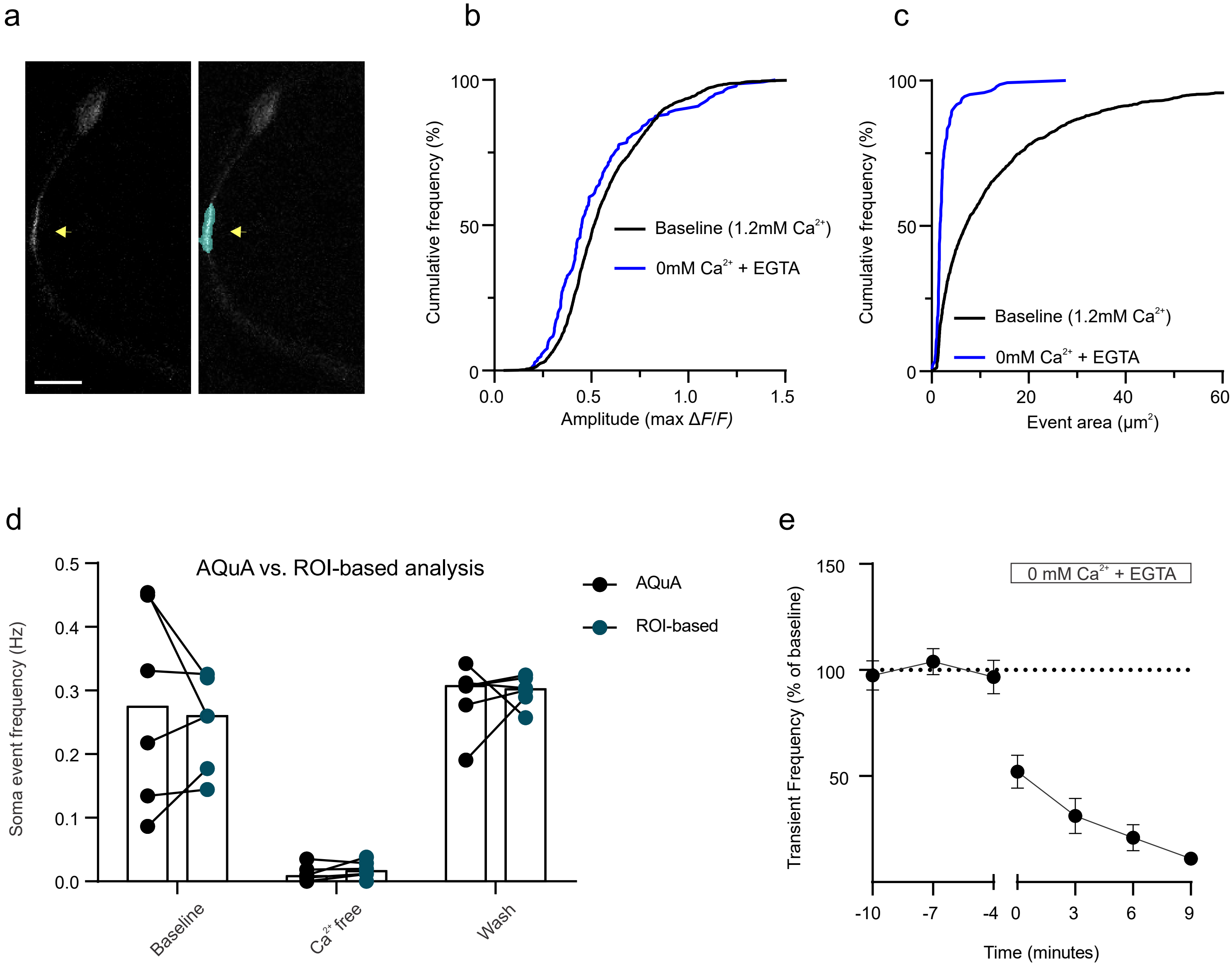

Supplementary Figure 1: Related to Fig. 1, Imaging of mid-capillary pericyte microdomain Ca^{2+} signals and their dependence on extracellular Ca^{2+}

(a) An example of an AQuA detected event in a mid-capillary pericyte process, taken from **Movie S1**. Scale bar = 10 µm.

(b, c) Cumulative histograms of event amplitude **(b)** and event area **(c)** at baseline (black) and following wash out of extracellular Ca^{2+} (blue), for 4-13 minutes. 1572 events (baseline), 153 events (0 mM Ca^{2+}), n = 6 cells.

(d) Comparison of event frequency measured in the soma using the AQuA event-based detection method to an ROI based analysis (> 3 standard deviations from baseline). Same cells analyzed in **Fig. 1e**.

(e) Time course of the effect of $[Ca^{2+}]_{ex}$ removal on the transient frequency in mid-capillary pericytes (processes + soma). n = 11 cells analyzed from **Fig. 1f-g** in which 50 second videos were taken every 3 minutes, x-axis represents measurements over a duration of 50 seconds starting at time, t. Time 0 represents time at which solution was calculated to reach the bath based on the flow rate, which was calculated to vary by +/- 30 seconds over the course of an experimental day.

Supplementary Figure 2

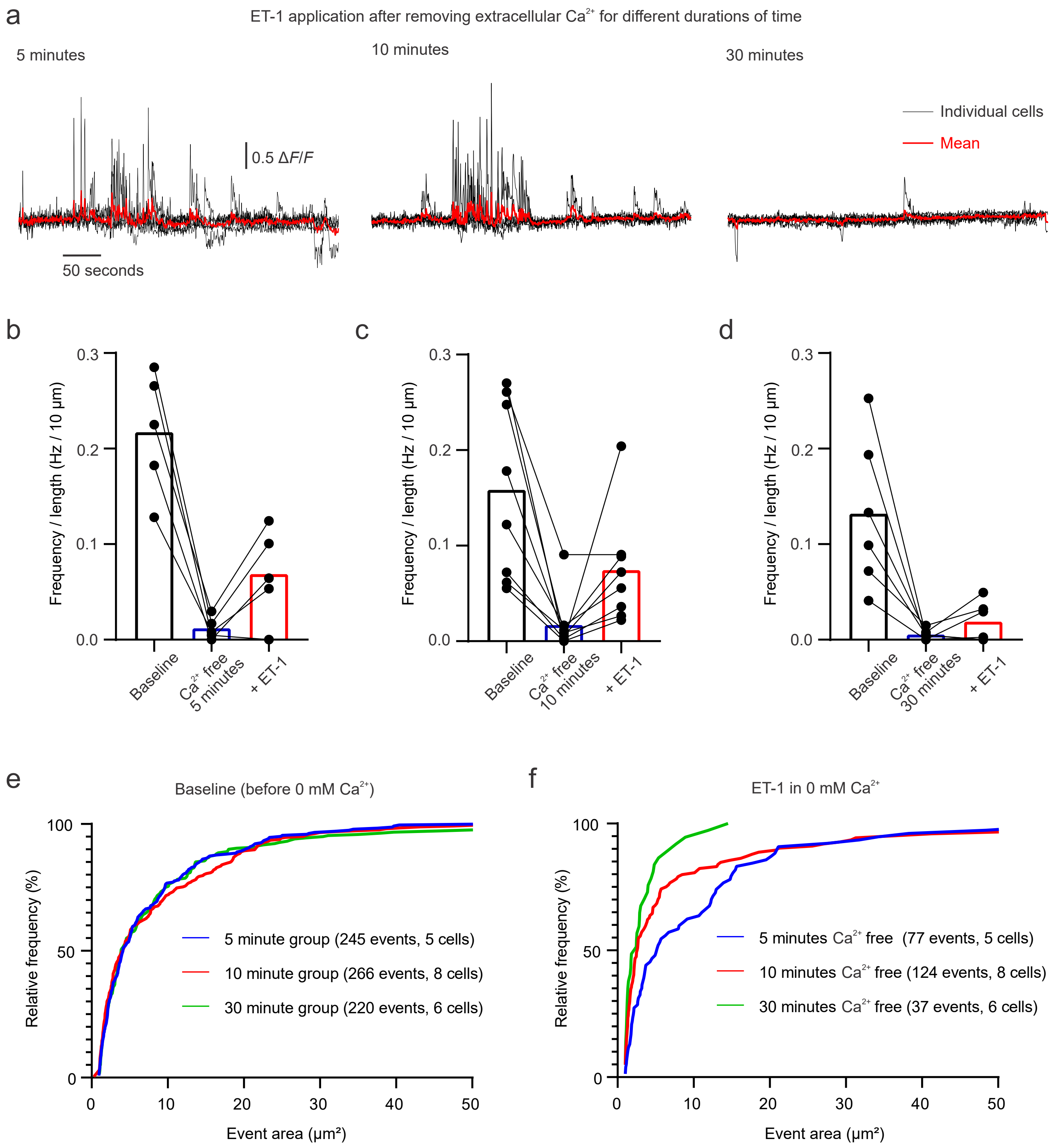

Supplementary Figure 2: Related to Fig. 1, Effect of ET-1 at different time points following extracellular Ca^{2+} removal.
(a) Example traces of GCaMP6f fluorescence in the soma of mid-capillary pericytes when ET-1 was applied either 5 minutes (left), 10 minutes (middle), or 30 minutes (right) following perfusion of ACSF containing 0 mM $[Ca^{2+}]_{ex}$, 2 mM EGTA. Black traces represent overlaid traces from 5 individual cells in each condition, red trace represents the mean of the 5 traces.

Legend continued on next page

(b-d) Summarized data showing reduction in Ca^{2+} transients in mid-capillary pericytes in response to 5 minutes (**b**), 10 minutes (**c**), and 30 minutes (**d**) of ACSF containing 0 mM $[Ca^{2+}]_{ex}$, 2 mM EGTA, and the effect of ET-1 on increasing transient frequency following these different time points.
(e-f) Cumulative histograms of event area, (**e**) shows that in control ACSF the distribution of event area was similar across the 3 groups, but that the Ca^{2+} transients evoked by ET-1 application had smaller event areas with increasing duration of extracellular Ca^{2+} removal. Analysis of event frequency in b-f, represents an analysis period of 50 seconds in each condition for each cell. N = 4 mice, n = 19 cells, 1 cell per brain slice.

Supplementary Figure 3

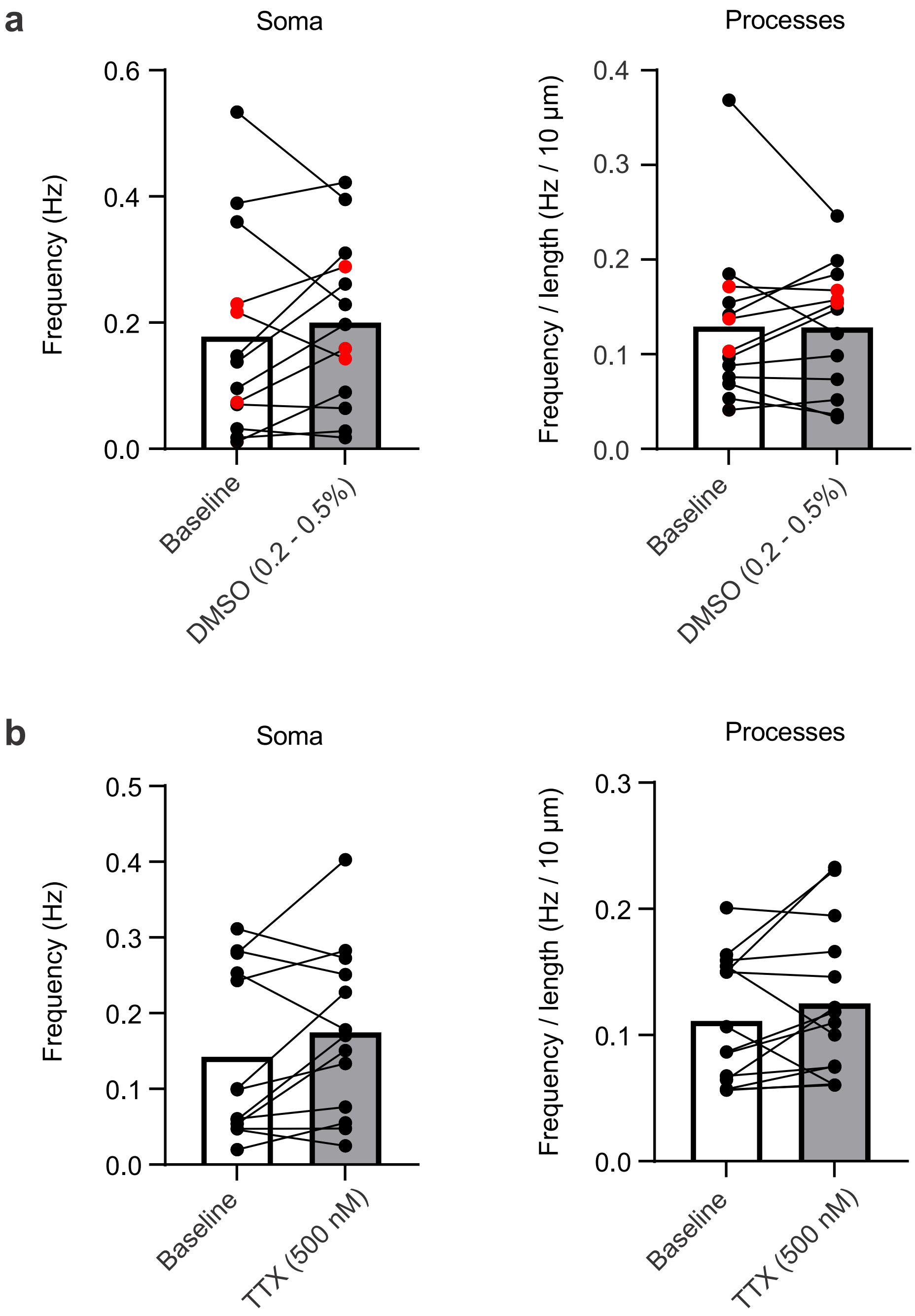

Supplementary Figure 3: Related to Fig. 2, Analysis of Ca^{2+} transient frequency in response to DMSO and 500 nM TTX.
(**a**) Vehicle control showing that 0.2% (black circles) - 0.5% (red circles) dimethyl sulfoxide (DMSO) used in this study does not significantly alter mid-capillary pericyte Ca^{2+} transient frequency in either the soma (left; N = 3, n = 13) or processes (right; N = 3, n = 13).
(**b**) Perfusion of 500 nM tetrodotoxin (TTX) onto brain slices to block neuronal action potentials did not significantly alter mid-capillary pericyte Ca^{2+} transient frequency in either the soma (left; N = 6, n = 13) or processes (right; N = 6, n = 14).

Supplementary Figure 4

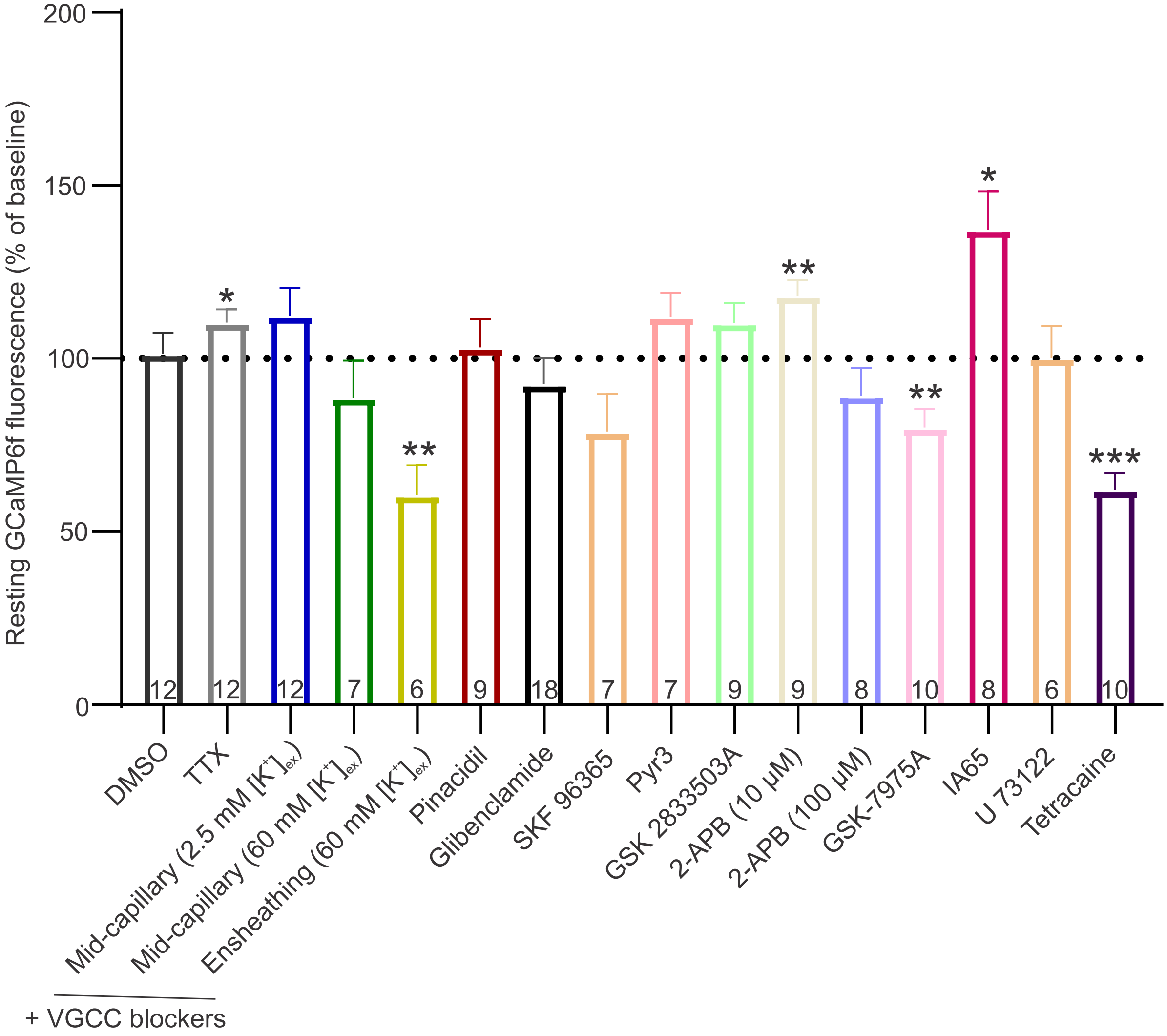

Supplementary Figure 4: Related to Fig. 2-4, Effects of pharmacological compounds on resting Ca^{2+}.
Analysis of changes in resting GCaMP6f fluorescence compared to baseline of various drugs used in the study. Measurements made in the soma (10-20 minutes after drug application). Numbers at bottom of bars represent n values (cells), Error bars represent SEM.

Supplementary Table 1: Summarized data and statistics for all pharmacological treatments on pericyte processes

Drug	Mice (N)	Cells (n)	Frequency (Hz / 10 µm)		
			Baseline	Drug	p-value
DMSO (0.2 - 0.5%)	3	13	0.1296 (± 0.02354)	0.1286 (± 0.01840)	0.9418
TTX (500 nM)	6	14	0.1114 (± 0.01330)	0.1252 (± 0.01626)	0.2028
Nifedipine (20 µM) and Z944 (2 µM)	2	16	0.153 (± 0.02303)	0.1646 (± 0.03816)	0.5926
Nifedipine (20 µM) and Z944 (2 µM) in 60mM K+ (mid-capillary)	4	8	0.1775 (± 0.02488)	0.1581 (± 0.02499)	0.1385
Nifedipine (20 µM) and Z944 (2 µM) in 60mM K+ (ensheathing)	5	9	0.1389 (± 0.01308)	0.05067 (± 0.01009)	**0.0019**
Pinacidil (10 µM)	2	11	0.1102 (± 0.02124)	0.09687 (± 0.01881)	0.2079
Glibenclamide (20 µM)	3	20	0.08559 (± 0.01257)	0.05062 (± 0.009259)	**0.0003**
SKF 963365 (100 µM)	3	8	0.1614 (± 0.03523)	0.04428 (± 0.01427)	**0.0133**
Pyr3 (20 µM)	3	7	0.1323 (± 0.02181)	0.1258 (± 0.01808)	0.6291
GSK 2833503A (10 µM)	2	9	0.191 (± 0.01208)	0.1703 (± 0.01043)	0.1183
2-APB (10 µM)	3	9	0.1279 (± 0.02547)	0.1591 (± 0.01953)	**0.0384**
2-APB (100 µM)	3	9	0.09411 (± 0.01592)	0.01759 (± 0.009440)	**0.0001**
GSK 7975A (40 µM)	2	10	0.1488 (± 0.02143)	0.06693 (± 0.01065)	**0.0001**
IA65 (10 µM)	2	8	0.1431 (± 0.03229)	0.1215 (± 0.03076)	0.1168
U 73122 (25 µM)	2	6	0.1667 (± 0.02476)	0.1180 (± 0.02093)	**0.0093**
Tetracaine (200 µM)	3	10	0.1741 (± 0.02332)	0.03721 (± 0.008163)	**0.0004**
60 mM K^+ (mid-capillary): *Processes plus soma analyzed (Fig. 2d)*	2	8	0.1989 (± 0.03499)	0.1449 (± 0.02388)	0.1715
60 mM K^+ (ensheathing): *Processes plus soma analyzed (Fig. 2d)*	2	21	0.04909 (± 0.009518)	0.1155 (± 0.01700)	**0.0001**

Drug	Mice (N)	Cells (n)	Area (µm^2)		
			Baseline	Drug	p-value
DMSO (0.2 - 0.5%)	3	13	9.213 (± 0.7387)	8.316 (± 0.6403)	0.1647
TTX (500 nM)	6	14	10.75 (± 1.039)	9.573 (± 0.7749)	0.3454
Nifedipine (20 µM) and Z944 (2 µM)	2	16	4.914 (± 0.5680)	4.286 (± 0.6428)	0.3258
Nifedipine (20 µM) and Z944 (2 µM) in 60mM K+ (mid-capillary)	4	8	14.07 (± 3.532)	11.83 (± 2.747)	0.1705
Nifedipine (20 µM) and Z944 (2 µM) in 60mM K+ (ensheathing)	5	9	14.18 (± 2.748)	10.02 (± 2.570)	**0.0081**
Pinacidil (10 µM)	2	11	4.994 (± 0.6130)	4.496 (± 0.5458)	0.2143
Glibenclamide (20 µM)	3	20	4.611 (± 0.7727)	3.244 (± 0.3403)	**0.0375**
SKF 963365 (100 µM)	3	8	14.84 (± 2.363)	5.190 (± 1.118)	**0.011**
Pyr3 (20 µM)	3	7	9.572 (± 0.9827)	9.040 (± 0.8545)	0.2371
GSK 2833503A (10 µM)	2	9	9.813 (± 1.588)	8.732 (± 1.456)	0.0815
2-APB (10 µM)	3	9	10.79 (± 1.151)	10.44 (± 0.9508)	0.6511
2-APB (100 µM)	3	9	9.418 (± 2.199)	3.761 (± 0.5282)	**0.0377**
GSK 7975A (40 µM)	2	10	9.672 (± 1.097)	7.393 (± 1.542)	**0.0328**
IA65 (10 µM)	2	8	12.54 (± 1.524)	7.835 (± 1.419)	**0.0221**
U 73122 (25 µM)	2	6	11.48 (± 2.409)	8.881 (± 1.543)	0.1232
Tetracaine (200 µM)	3	10	10.61 (± 1.055)	6.027 (± 1.017)	**0.0001**
60 mM K^+ (mid-capillary): *Processes plus soma analyzed (Fig. 2d)*	2	8	13.40 (± 2.943)	9.987 (± 1.097)	0.272
60 mM K^+ (ensheathing): *Processes plus soma analyzed (Fig. 2d)*	2	21	8.053 (± 1.692)	7.795 (± 1.344)	0.8197

Drug	Mice (N)	Cells (n)	Amplitude (dF/F)		
			Baseline	Drug	p-value
DMSO (0.2 - 0.5%)	3	13	1.362 (± 0.06796)	1.299 (± 0.05628)	0.0559
TTX (500 nM)	6	14	1.275 (± 0.07252)	1.217 (± 0.07313)	0.2886
Nifedipine (20 µM) and Z944 (2 µM)	2	16	1.26 (± 0.06352)	1.401 (± 0.1762)	0.4526
Nifedipine (20 µM) and Z944 (2 µM) in 60mM K+ (mid-capillary)	4	8	1.314 (± 0.09787)	1.242 (± 0.06909)	0.2448
Nifedipine (20 µM) and Z944 (2 µM) in 60mM K+ (ensheathing)	5	9	1.136 (± 0.06587)	2.640 (± 1.459)	0.3378
Pinacidil (10 µM)	2	11	1.452 (± 0.1140)	1.407 (± 0.1032)	0.1515
Glibenclamide (20 µM)	3	20	1.424 (± 0.05288)	1.531 (± 0.07824)	0.1615
SKF 963365 (100 µM)	3	8	1.554 (± 0.1869)	1.419 (± 0.1046)	0.262
Pyr3 (20 µM)	3	7	1.468 (± 0.06324)	1.41 (± 0.02775)	0.2821
GSK 2833503A (10 µM)	2	9	1.147 (± 0.08309)	1.108 (± 0.07834)	0.1069
2-APB (10 µM)	3	9	1.319 (± 0.07848)	1.158 (± 0.05173)	**0.0018**
2-APB (100 µM)	3	9	1.309 (± 0.1818)	1.288 (± 0.1376)	0.9244
GSK 7975A (40 µM)	2	10	1.156 (± 0.07688)	1.191 (± 0.05333)	0.3832
IA65 (10 µM)	2	8	1.253 (± 0.07170)	1.204 (± 0.06083)	0.2333
U 73122 (25 µM)	2	6	1.314 (± 0.08654)	1.426 (± 0.1775)	0.511
Tetracaine (200 µM)	3	10	1.196 (± 0.0627)	1.574 (± 0.1147)	**0.014**
60 mM K^+ (mid-capillary): *Processes plus soma analyzed (Fig. 2d)*	2	8	1.154 (± 0.1302)	1.316 (± 0.03653)	0.2116
60 mM K^+ (ensheathing): *Processes plus soma analyzed (Fig. 2d)*	2	21	1.047 (± 0.07867)	1.382 (± 0.3249)	0.3132

Drug	Mice (N)	Cells (n)	Duration (s)		
			Baseline	Drug	p-value
DMSO (0.2 - 0.5%)	3	13	1.537 (± 0.06649)	1.489 (± 0.08689)	0.3724
TTX (500 nM)	6	14	1.725 (± 0.05649)	1.696 (± 0.05343)	0.5653
Nifedipine (20 µM) and Z944 (2 µM)	2	16	1.416 (± 0.06997)	1.483 (± 0.1627)	0.6518
Nifedipine (20 µM) and Z944 (2 µM) in 60mM K+ (mid-capillary)	4	8	1.439 (± 0.04916)	1.339 (± 0.05710)	0.084
Nifedipine (20 µM) and Z944 (2 µM) in 60mM K+ (ensheathing)	5	9	1.699 (± 0.08994)	1.827 (± 0.4185)	0.7927
Pinacidil (10 µM)	2	11	1.372 (± 0.07622)	1.234 (± 0.05032)	0.0796
Glibenclamide (20 µM)	3	20	1.147 (± 0.04976)	1.099 (± 0.03433)	0.3368
SKF 963365 (100 µM)	3	8	1.925 (± 0.2676)	1.404 (± 0.2933)	**0.0314**
Pyr3 (20 µM)	3	7	1.613 (± 0.07212)	1.582 (± 0.06962)	0.6469
GSK 2833503A (10 µM)	2	9	1.65 (± 0.06381)	1.547 (± 0.04660)	0.1074
2-APB (10 µM)	3	9	1.538 (± 0.05469)	1.548 (± 0.03383)	0.7812
2-APB (100 µM)	3	9	1.654 (± 0.1066)	1.289 (± 0.1475)	0.1332
GSK 7975A (40 µM)	2	10	1.787 (± 0.04214)	1.451 (± 0.07618)	**0.0004**
IA65 (10 µM)	2	8	1.573 (± 0.0429)	1.640 (± 0.05330)	0.2181
U 73122 (25 µM)	2	6	1.575 (± 0.09196)	1.675 (± 0.1132)	0.1901
Tetracaine (200 µM)	3	10	1.453 (± 0.06113)	1.172 (± 0.05666)	**0.0038**
60 mM K^+ (mid-capillary): *Processes plus soma analyzed (Fig. 2d)*	2	8	1.400 (± 0.06776)	1.572 (± 0.06427)	0.1018
60 mM K^+ (ensheathing): *Processes plus soma analyzed (Fig. 2d)*	2	21	1.351 (± 0.05639)	1.870 (± 0.1144)	**0.0002**

Supplementary Table 2: Summarized data and statistics for all pharmacological treatments on pericyte soma

Drug	Mice (N)	Cells (n)	Frequency (Hz)			Resting Ca2+ (% of baseline GCaMP6f fluoresence)	
			Baseline	Drug	p-value	Mean	p-value
DMSO (0.2 - 0.5%)	3	13	0.1781 (± 0.04495)	0.2004 (± 0.03673)	0.4084	101.5 (± 5.946)	0.8057
TTX (500 nM)	6	13	0.1427 (± 0.03076)	0.1749 (± 0.03067)	0.1047	110.4 (± 3.853)	**0.0194**
Nifedipine (20 µM) and Z944 (2 µM)	2	16	0.3039 (± 0.03947)	0.3517 (± 0.04741)	0.0593	112.44 (± 8.032)	0.1511
Nifedipine (20 µM) and Z944 (2 µM) in 60mM K+ (mid-capillary)	4	8	0.4284 (± 0.05747)	0.4196 (± 0.08973)	0.8977	88.79 (± 10.62)	0.3318
Nifedipine (20 µM) and Z944 (2 µM) in 60mM K+ (ensheathing)	5	6	0.3068 (± 0.04269)	0.09877 (± 0.03751)	**0.0158**	60.56 (± 8.675)	**0.0061**
Pinacidil (10 µM)	2	11	0.171 (± 0.05009)	0.1723 (± 0.05375)	0.9387	103.3 (± 8.077)	0.6971
Glibenclamide (20 µM)	3	15	0.1624 (± 0.03490)	0.1305 (± 0.03253)	0.3809	92.54 (± 7.665)	0.3441
SKF 963365 (100 µM)	3	8	0.1951 (± 0.05209)	0.06131 (± 0.02653)	**0.0291**	78.89 (± 10.85)	0.0997
Pyr3 (20 µM)	3	7	0.1922 (± 0.02889)	0.1965 (± 0.03481)	0.9179	112 (± 7.099)	0.1414
GSK 2833503A (10 µM)	2	9	0.3298 (± 0.03407)	0.2852 (± 0.04663)	0.1583	110.3 (± 5.760)	0.1106
2-APB (10 µM)	3	9	0.1643 (± 0.03763)	0.2565 (± 0.03821)	**0.0069**	118.2 (± 4.591)	**0.0042**
2-APB (100 µM)	3	8	0.1406 (± 0.04465)	0.03530 (± 0.009946)	**0.0344**	89.25 (± 7.937)	0.2176
GSK 7975A (40 µM)	2	10	0.2499 (± 0.02938)	0.1321 (± 0.02374)	**0.0011**	80.09 (± 5.310)	**0.0046**
IA65 (10 µM)	2	7	0.2260 (± 0.06223)	0.2051 (± 0.03479)	0.685	137.3 (± 10.97)	**0.0114**
U 73122 (25 µM)	2	6	0.2622 (± 0.04338)	0.2529 (± 0.03615)	0.7216	100.3 (± 9.128)	0.9786
Tetracaine (200 µM)	3	10	0.2302 (± 0.05449)	0.04226 (± 0.01326)	**0.0054**	62.08 (± 4.770)	**0.0001**

Drug	Mice (N)	Cells (n)	Area (µm^2)		
			Baseline	Drug	p-value
DMSO (0.2 - 0.5%)	3	13	13.12 (± 2.250)	11.76 (± 1.592)	0.5178
TTX (500 nM)	6	13	13.31 (± 2.112)	14.00 (± 2.713)	0.7843
Nifedipine (20 µM) and Z944 (2 µM)	2	16	5.81 (± 0.7784)	7.436 (± 1.444)	0.0683
Nifedipine (20 µM) and Z944 (2 µM) in 60mM K+ (mid-capillary)	4	8	27.83 (± 7.515)	21.25 (± 5.037)	0.1618
Nifedipine (20 µM) and Z944 (2 µM) in 60mM K+ (ensheathing)	5	6	28.51 (± 4.200)	9.546 (± 3.793)	**0.0032**
Pinacidil (10 µM)	2	11	5.84 (± 1.262)	5.161 (± 1.084)	0.3919
Glibenclamide (20 µM)	3	15	3.845 (± 0.6481)	2.740 (± 0.3747)	0.102
SKF 963365 (100 µM)	3	8	19.29 (± 5.849)	4.230 (± 1.210)	**0.0438**
Pyr3 (20 µM)	3	7	11.79 (± 2.204)	10.78 (± 2.546)	0.5794
GSK 2833503A (10 µM)	2	9	16.81 (± 3.626)	15.15 (± 3.835)	0.468
2-APB (10 µM)	3	9	17.89 (± 3.149)	14.8 (± 1.193)	0.2821
2-APB (100 µM)	3	8	12.7 (± 1.677)	2.841 (± 0.7122)	**0.0023**
GSK 7975A (40 µM)	2	10	14.65 (± 1.635)	12.23 (± 2.111)	0.1935
IA65 (10 µM)	2	7	17.42 (± 4.010)	11.54 (± 3.348)	0.0696
U 73122 (25 µM)	2	6	18.28 (± 3.822)	9.084 (± 1.315)	**0.0265**
Tetracaine (200 µM)	3	10	16.04 (± 3.815)	3.525 (± 0.9906)	**0.0078**

Drug	Mice (N)	Cells (n)	Amplitude (dF/F)		
			Baseline	Drug	p-value
DMSO (0.2 - 0.5%)	3	13	1.227 (± 0.07934)	1.196 (± 0.07938)	0.7455
TTX (500 nM)	6	13	1.235 (± 0.09715)	1.133 (± 0.08491)	0.3214
Nifedipine (20 µM) and Z944 (2 µM)	2	16	1.016 (± 0.05588)	1.203 (± 0.2211)	0.4032
Nifedipine (20 µM) and Z944 (2 µM) in 60mM K+ (mid-capillary)	4	8	1.211 (± 0.1599)	1.063 (± 0.1085)	0.2052
Nifedipine (20 µM) and Z944 (2 µM) in 60mM K+ (ensheathing)	5	6	1.059 (± 0.08104)	1.066 (± 0.1306)	0.9537
Pinacidil (10 µM)	2	11	1.297 (± 0.1370)	1.039 (± 0.07270)	0.1108
Glibenclamide (20 µM)	3	15	1.098 (± 0.08155)	1.009 (± 0.08089)	0.0891
SKF 963365 (100 µM)	3	8	1.687 (± 0.3553)	1.116 (± 0.1398)	0.1283
Pyr3 (20 µM)	3	7	1.332 (± 0.1250)	1.219 (± 0.08436)	0.1697
GSK 2833503A (10 µM)	2	9	1.042 (± 0.1199)	0.9326 (± 0.1037)	0.2648
2-APB (10 µM)	3	9	1.294 (± 0.1663)	1.017 (± 0.07862)	**0.0421**
2-APB (100 µM)	3	8	1.205 (± 0.1632)	0.7631 (± 0.04756)	**0.036**
GSK 7975A (40 µM)	2	10	1.079 (± 0.1129)	1.153 (± 0.1282)	0.2615
IA65 (10 µM)	2	7	1.051 (± 0.1301)	0.9300 (± 0.06816)	0.3818
U 73122 (25 µM)	2	6	1.079 (± 0.0843)	0.9697 (± 0.03612)	0.1801
Tetracaine (200 µM)	3	10	0.9379 (± 0.06413)	1.160 (± 0.1243)	0.2096

Drug	Mice (N)	Cells (n)	Duration (s)		
			Baseline	Drug	p-value
DMSO (0.2 - 0.5%)	3	13	1.680 (± 0.2046)	1.679 (± 0.1288)	0.9981
TTX (500 nM)	6	13	1.812 (± 0.1669)	1.838 (± 0.09642)	0.8933
Nifedipine (20 µM) and Z944 (2 µM)	2	16	1.562 (± 0.07127)	1.652 (± 0.1180)	0.4504
Nifedipine (20 µM) and Z944 (2 µM) in 60mM K+ (mid-capillary)	4	8	1.678 (± 0.06614)	1.505 (± 0.08723)	0.1315
Nifedipine (20 µM) and Z944 (2 µM) in 60mM K+ (ensheathing)	5	6	1.843 (± 0.07871)	1.299 (± 0.2078)	**0.0223**
Pinacidil (10 µM)	2	11	1.408 (± 0.1344)	1.339 (± 0.09024)	0.589
Glibenclamide (20 µM)	3	15	1.346 (± 0.09424)	1.155 (± 0.07388)	0.0538
SKF 963365 (100 µM)	3	8	1.925 (± 0.2676)	1.404 (± 0.2933)	**0.0314**
Pyr3 (20 µM)	3	7	1.778 (± 0.09761)	1.525 (± 0.1765)	0.0806
GSK 2833503A (10 µM)	2	9	1.887 (± 0.1064)	1.853 (± 0.09920)	0.8186
2-APB (10 µM)	3	9	1.820 (± 0.1660)	1.747 (± 0.07129)	0.6283
2-APB (100 µM)	3	8	2.073 (± 0.3742)	1.587 (± 0.3125)	0.1266
GSK 7975A (40 µM)	2	10	2.094 (± 0.1152)	2.033 (± 0.1019)	0.7195
IA65 (10 µM)	2	7	1.848 (± 0.2062)	2.015 (± 0.1478)	0.167
U 73122 (25 µM)	2	6	1.829 (± 0.1461)	1.776 (± 0.1065)	0.7665
Tetracaine (200 µM)	3	10	1.607 (± 0.1644)	1.256 (± 0.1302)	0.1018

Supplementary Table 3: List of pharmacological compounds, intended targets, vehichles.

	Drug (concentration used)														
**Target**	Nifedipine (20 µM)	Z944 (2 µM)	Pinacidil (10 µM)	Glibenclamide (20 µM)	SKF-96365 (100 µM)	Pyr3 (20 µM)	GSK 2833503A (10 µM)	2-APB (10 - 100 µM)	GSK 7975A (40 µM)	IA65 (10 µM)	CPA (30 µM)	U 73122 (25 µM)	Tetracaine (200 µM)	Caffeine (10 mM)	Ani9 (2 µM)
CACNA1C	225 nM [1]								8 µM [16]						
CACNA1H		50 nM [2]			563 nM [4]				> 10 µM * [16]						
KCNJ8 (KATP)			EC_{50} = 600 nM [3]	101 nM [3]											
TRPC3					5 µM [5]	700 nM [9]	100 nM [10]	60% inhibition at 30 µM [11]	> 10 µM * [16]						
TRPC6					4 µM [6]		16 nM [10]	Complete inhibition at 75 µM [12]	> 10 µM * [16]						
Orai1					4 - 12 µM [7,8]			EC_{50} = 3 µM; IC_{50} = 10 µM [13]	4.1 µM [16]	EC_{50} = 1.9 µM [17]					
Orai3					4 - 12 µM [7,8]			EC_{50} = 24 µM [14]	3.8 µM [16]	Potentiated at 10 µM[18]					
SERCA ATPases											3.2 µM [19]				
IP3R								42 µM [15]							
Phospholipase C												2 - 4 µM [20, 21]			
RyR													200 µM ** [22]	EC_{50}: 0.4 - 9.2 mM *** [23]	
TMEM16A (ANO1)															77 nM [24]

Concentrations represent drug IC50 unless otherwise stated.

* No observed effect of GSK-7975A at 10 µM on these targets

** Dependent on voltage and levels of intracellular ATP, cytosolic Ca2+, and luminal Ca2+

*** Dependent on levels of cytosolic and luminal Ca2+

Drug	Supplier	Identifier	Vehicle (concentration in solution)
TO-PRO-3	Thermo Scientific	T3605	DMSO (0.1%)
EGTA	Sigma-Aldrich	E3889	N/A
U46619	Tocris	Cat. No. 1932	H_2O (0.01%)
Endothelin-1	Millipore Sigma	05-23-3800	H_2O (0.1%)
Tetrodotoxin citrate	Abcam	ab120055	H_2O (0.05%)
Nifedipine	Sigma-Aldrich	N7634	DMSO (0.1%)
Z944	Dr. Terrance Snutch, University of British Columbia		DMSO (0.02%)
Pinacidil monohydrate	Sigma-Aldrich	P154	DMSO (0.1%)
Glibenclamide	Tocris	Cat. No. 0911	DMSO (0.1%)
SKF 96365 hydrochloride	Tocris	Cat. No. 1147	H_2O (0.5%)
Pyr3	Tocris	Cat. No. 3751	DMSO (0.1%)
GSK 2833503A	Tocris	Cat. No. 6497	DMSO (0.1%)
2-APB	Millipore Sigma	100065	DMSO (0.02% or 0.2%)
GSK-7975A	AdooQ BioScience	A16931-10	DMSO (0.1%)
Cyclopiazonic acid	Tocris	Cat. No. 1235	DMSO (0.1%)
IA65	Dr. Mohamed Trebak, University of Pittsburgh		DMSO (0.02%)
U 73122	Tocris	Cat. No. 1268	DMSO (0.5%)
Tetracaine hydrochloride	Sigma-Aldrich	T7508	H_2O (0.2%)
Caffeine	Sigma-Aldrich	C0750	N/A

References for Table S3

1. Bradley JE, Anderson UA, Woolsey SM, Thornbury KD, McHale NG, Hollywood MA (2004) Characterization of T-type calcium current and its contribution to electrical activity in rabbit urethra. American Journal of Physiology-Cell Physiology 286:C1078-C1088.
2. Tringham E, Powell KL, Cain SM, Kuplast K, Mezeyova J, Weerapura M, Eduljee C, Jiang X, Smith P, Morrison J-L, Jones NC, Braine E, Rind G, Fee-Maki M, Parker D, Pajouhesh H, Parmar M, O'Brien TJ, Snutch TP (2012) T-Type Calcium Channel Blockers That Attenuate Thalamic Burst Firing and Suppress Absence Seizures. Science Translational Medicine 4:121ra119-121ra119.
3. Quayle JM, Bonev AD, Brayden JE, Nelson MT (1995) Pharmacology of ATP-sensitive K+ currents in smooth muscle cells from rabbit mesenteric artery. American Journal of Physiology-Cell Physiology 269:C1112-C1118.
4. Singh A, Hildebrand M, Garcia E, Snutch T (2010) The transient receptor potential channel antagonist SKF96365 is a potent blocker of low-voltage-activated T-type calcium channels. British Journal of Pharmacology 160:1464-1475.
5. Zhu X, Jiang M, Birnbaumer L (1998) Receptor-activated Ca2+ influx via human Trp3 stably expressed in human embryonic kidney (HEK)293 cells. Evidence for a non-capacitative Ca2+ entry. J Biol Chem 273:133-142.
6. Inoue R, Okada T, Onoue H, Hara Y, Shimizu S, Naitoh S, Ito Y, Mori Y (2001) The Transient Receptor Potential Protein Homologue TRP6 Is the Essential Component of Vascular α₁-Adrenoceptor–Activated Ca²⁺-Permeable Cation Channel. Circulation Research 88:325-332.
7. Franzius D, Hoth M, Penner R (1994) Non-specific effects of calcium entry antagonists in mast cells. Pflugers Arch 428:433-438.
8. Chung SC, McDonald TV, Gardner P (1994) Inhibition by SK&F 96365 of Ca2+ current, IL-2 production and activation in T lymphocytes. British Journal of Pharmacology 113:861-868.
9. Kiyonaka S et al. (2009) Selective and direct inhibition of TRPC3 channels underlies biological activities of a pyrazole compound. Proc Natl Acad Sci U S A 106:5400-5405.
10. Washburn DG et al. (2013) The discovery of potent blockers of the canonical transient receptor channels, TRPC3 and TRPC6, based on an anilino-thiazole pharmacophore. Bioorg Med Chem Lett 23:4979-4984.
11. Trebak M, Bird GS, McKay RR, Putney JW, Jr. (2002) Comparison of human TRPC3 channels in receptor-activated and store-operated modes. Differential sensitivity to channel blockers suggests fundamental differences in channel composition. J Biol Chem 277:21617-21623.
12. Xu SZ, Zeng F, Boulay G, Grimm C, Harteneck C, Beech DJ (2005) Block of TRPC5 channels by 2-aminoethoxydiphenyl borate: a differential, extracellular and voltage-dependent effect. Br J Pharmacol 145:405-414.
13. Prakriya M, Lewis RS (2001) Potentiation and inhibition of Ca(2+) release-activated Ca(2+) channels by 2-aminoethyldiphenyl borate (2-APB) occurs independently of IP(3) receptors. J Physiol 536:3-19.
14. Yamashita M, Prakriya M (2014) Divergence of Ca2+ selectivity and equilibrium Ca2+ blockade in a Ca2+ release-activated Ca2+ channel. Journal of General Physiology 143:325-343.

15. Maruyama T, Kanaji T, Nakade S, Kanno T, Mikoshiba K (1997) 2APB, 2-aminoethoxydiphenyl borate, a membrane-penetrable modulator of Ins(1,4,5)P3-induced Ca2+ release. J Biochem 122:498-505.
16. Derler I, Schindl R, Fritsch R, Heftberger P, Riedl MC, Begg M, House D, Romanin C (2013) The action of selective CRAC channel blockers is affected by the Orai pore geometry. Cell Calcium 53:139-151.
17. Azimi I, Stevenson RJ, Zhang X, Meizoso-Huesca A, Xin P, Johnson M, Flanagan JU, Chalmers SB, Yoast RE, Kapure JS, Ross BP, Vetter I, Ashton MR, Launikonis BS, Denny WA, Trebak M, Monteith GR (2020) A new selective pharmacological enhancer of the Orai1 Ca(2+) channel reveals roles for Orai1 in smooth and skeletal muscle functions. ACS Pharmacol Transl Sci 3:135-147.
18. Zhang X, Xin P, Yoast RE, Emrich SM, Johnson MT, Pathak T, Benson C, Azimi I, Gill DL, Monteith GR, Trebak M (2020) Distinct pharmacological profiles of ORAI1, ORAI2, and ORAI3 channels. Cell Calcium 91: 102281.
19. Demaurex N, Lew DP, Krause KH (1992) Cyclopiazonic acid depletes intracellular Ca2+ stores and activates an influx pathway for divalent cations in HL-60 cells. J Biol Chem 267:2318-2324.
20. Smith RJ, Sam LM, Justen JM, Bundy GL, Bala GA, Bleasdale JE (1990) Receptor-coupled signal transduction in human polymorphonuclear neutrophils: effects of a novel inhibitor of phospholipase C-dependent processes on cell responsiveness. J Pharmacol Exp Ther 253:688-697.
21. Thompson AK, Mostafapour SP, Denlinger LC, Bleasdale JE, Fisher SK (1991) The aminosteroid U-73122 inhibits muscarinic receptor sequestration and phosphoinositide hydrolysis in SK-N-SH neuroblastoma cells. A role for Gp in receptor compartmentation. J Biol Chem 266:23856-23862.
22. Laver DR, van Helden DF (2011) Three independent mechanisms contribute to tetracaine inhibition of cardiac calcium release channels. J Mol Cell Cardiol 51:357-369.
23. Porta M, Zima AV, Nani A, Diaz-Sylvester PL, Copello JA, Ramos-Franco J, Blatter LA, Fill M (2011) Single ryanodine receptor channel basis of caffeine's action on Ca2+ sparks. Biophys J 100:931-938.
24. Seo Y, Lee HK, Park J, Jeon DK, Jo S, Jo M, Namkung W (2016) Ani9, A Novel Potent Small-Molecule ANO1 Inhibitor with Negligible Effect on ANO2. PLoS One 11:e0155771.
