# Supplementary material for: Orai, RyR, and IP3R channels cooperatively regulate calcium signaling in brain mid-capillary pericytes
Source: Commun Biol. 2023 May 6;6:493. doi: 10.1038/s42003-023-04858-3 (PMC10164186; doi:10.1038/s42003-023-04858-3)
Supplement: Supplementary file 2 — Description of Additional Supplementary Data [file 42003_2023_4858_MOESM2_ESM.pdf]

Description of Additional Supplementary Files

File name: Supplementary Movie 1

Description: Related to Fig. 1, the movie shows GCaMP6f fluorescence over a 50 second recording of a thin strand pericyte (left), and colour coded identification of events detected by AQuA (right).

File name: Supplementary Data 1

Description: The source data behind the graphs in the paper.
